# Scheffe’s Simplex Optimization of Flexural Strength of Quarry Dust and Sawdust Ash Pervious Concrete for Sustainable Pavement Construction

**DOI:** 10.3390/ma16020598

**Published:** 2023-01-07

**Authors:** Desmond E. Ewa, Joseph O. Ukpata, Obeten Nicholas Otu, Zubair Ahmed Memon, George Uwadiegwu Alaneme, Abdalrhman Milad

**Affiliations:** 1 Department of Civil Engineering, University of Cross River State, Calabar 540271, Nigeria; 2 Department of Engineering Management, College of Engineering, Prince Sultan University (PSU), Riyadh 11586, Saudi Arabia; 3 Department of Civil Engineering, Kampala International University, Kampala 20000, Uganda; 4 Department of Civil Engineering, Michael Okpara University of Agriculture, Umudike, P. M. B. 7267, Umuahia 440109, Nigeria; 5 Department of Civil and Environmental Engineering, College of Engineering, University of Nizwa, Nizwa P.O. Box 33, Oman

**Keywords:** pervious concrete, flexural strength, microstructural and morphological assessments, abrasion resistance

## Abstract

Pervious concrete provides a tailored surface course with high permeability properties which permit the easy flow of water through a larger interconnected porous structure to prevent flooding hazards. This paper reports the modeling of the flexural properties of quarry dust (QD) and sawdust ash (SDA) blended green pervious concrete for sustainable road pavement construction using Scheffe’s (5,2) optimization approach. The simplex mixture design method was adapted to formulate the mixture proportion to eliminate the set-backs encountered in empirical or trials and the error design approach, which consume more time and resources to design with experimental runs required to evaluate the response function. For the laboratory evaluation exercise, a maximum flexural strength of 3.703 N/mm^2^ was obtained with a mix proportion of 0.435:0.95:0.1:1.55:0.05 for water, cement, QD, coarse aggregate and SDA, respectively. Moreover, the minimal flexural strength response of 2.504 N/mm^2^ was obtained with a mix ratio of 0.6:0.75:0.3:4.1:0.25 for water, cement, QD, coarse aggregate and SDA, respectively. The test of the appropriateness of the developed model was statistically verified using the Student’ *t*-test and an analysis of variance (ANOVA), and was confirmed to be acceptable based on computational outcomes at the 95% confidence interval. Furthermore, the scanning electron microscopy (SEM) and energy dispersive X-ray (EDX) were used to evaluate the morphological and mineralogical behavior of green prior concrete samples with various additive mixture compositions. The addition of QD and SDA, on the other hand, aided the creation of porous microstructures in the concrete matrix due to fabric changes in the concrete mixture, potentially aided by the formation of cementitious compounds such as calcium aluminate hydrate and calcium silicate hydrate.

## 1. Introduction

A pervious concrete is achieved by carefully controlling the quantity of water and cementitious materials to generate a thick coating paste with a substantial void content and highly permeable and interconnecting voids that can drain the surface runoff very quickly, which thereby preserves the service life of road pavement [1,2]. Pervious concrete applications for road pavement provide an effective and unique approach to address important environmental challenges in support of sustainable and green infrastructural development by providing a systematic technique to capture and convey storm water, allowing it to seep into the ground [3,4]. It is also one of the best environmental management practices recommended by the Environmental Protection Agency (EPA) for the adequate discharge of runoff storm water. The deployment of this advanced pavement drainage technology provides a more efficient use of land system by a total reduction in the need for swales, retention ponds and several other devices for the management of storm water [5]. Pervious concrete has a larger particle size with small finer aggregates that allow water from precipitation to permeate through them, thereby minimizing the runoff waters that may result in issues of flooding and erosion. It is a most essential application to achieve sustainable road pavement construction as well as a low-impact development methodology adapted to conserve and recharge groundwater [6,7]. The mixture generally possesses a water–cement ratio of 0.28–0.4, a coarse aggregate particle size range of 9.5–12.5 mm and a void content of 15–35%. The flexural strength of the concrete material helps to guide designers and engineers perform the safest and most efficient application [8].

Pervious concrete is known for being gap-graded and having enhanced porosity, whilst no fines concrete provides an innovative approach to the management, controlling and proper channeling of storm water runoff to a safe discharge point [9]. It is utilized in pavement applications to effectively discharge of and direct surface runoff by allowing its percolation through the ground to recharge groundwater. It has little or no fine aggregates with permissible quantities of cementitious materials and water [10]. Several research activities has been conducted in the area of the deployment of pervious concrete to achieve sustainable road pavements, which have contributed to reducing the risk of flooding. Ivana et al. [11] investigated the optimization of a pervious concrete mixture for sustainable pavement purposes by incorporating the new constituent’s materials (crushed dolomite) prepared with the variation distribution of aggregate fractions in a four-component mixture. The obtained laboratory results indicated that the pervious concrete with a single-sized aggregate mixture produced a higher porosity response with decreased strength properties, while the maximized share of coarse aggregate in the mixture is 40.21% whilst that of fine aggregate was 49.79 to give the required compressive strength of 25 Mpa, a porosity of 21.66% and a flexural strength of 4.31 Mpa. Marek and Alena [12] conducted an experimental investigation into pervious concrete as an advanced pavement material as an approach to finding an environmental solution. The permeability, void content, density under dry conditions as well as the compressive and splitting tensile strength properties were assessed for the concrete prepared with varying water–cement ratios (w/c) with the same volume of cement paste to derive a void content of 20%. Due to w/c variations of 0.25–0.35 and hydraulic gradient results of 7.5–10.2 mm/s, the acquired experimental findings demonstrated a minor effect on the mechanical strength behavior of the pervious concrete specimens.

Since the US Environmental Protection Agency (EPA) expanded storm water management requirements, there has been a demand for pavements that reduce surface runoff. Traditional roadway construction frequently fails to efficiently manage storm water runoff. Pervious concrete could be a viable solution to the problem of storm water runoff [13]. Pervious concrete’s high level of linked macro-porosity significantly reduces runoff from paved areas. Pervious concrete also has a number of other advantages. Pervious concrete, for example, is quieter to drive on than regular pavement because the porous surface absorbs sound. Pervious concrete can absorb storm water faster than ordinary concrete, resulting in better skid resistance [14]. The cementitious portion is substituted by wood waste derivatives to produce green concrete with higher durability potential. Several attempts to optimize the concrete design combination have been undertaken during the course, utilizing either empirical or analytical and statistical methodologies [15]. Empirical approaches entail a long series of tests, which are frequently performed by trial and error, and the optimization results are often limited to a small number of local materials. Consecutively, the number of exhaustive trials to be reduced before the optimum combination was established, and the use of analytical and statistical techniques would enhance the rationalizing of the initial trial mixes into a logical and analytical process [16,17]. This statistical method is very helpful in tracing of the optimum mixture combination based on the details of explicit weight functions of the combination cogs and basic formula resulting from prior experimental experiences without engaging in laborious, time-consuming and expensive work [18]. Scheffe’s simplex lattice approach is used to formulate and design the mix ratio of the ingredients in an attempt to optimize the concrete’s mechanical properties [19,20].

In related research literature works, Ubachukwu and Okafor [21] carried out an empirical study to develop and validate a predictive model using Scheffe’s simplex lattice method to evaluate oyster shell powder (OSP) concrete’s compressive strength behavior. From the experimental program carried out in this research work, a maximum compressive strength of 30.81 N/mm^2^ was obtained with a mix of 0.54:0.815:2.045:3.425:0.185 for water, cement, fine aggregate, coarse aggregate and OSP, respectively, while a minimum strength response of 17.85 N/mm^2^ was obtained with a mix of 0.525:0.825:2.2:4.05:0.175 for water, cement, fine aggregate, coarse aggregate and OSP, respectively. Alaneme and Mbadike [22] investigated the use of Scheffe’s theory to improve the flexural strength of concrete mixed with agricultural waste such as palm nut fiber. Cement, water, coarse aggregates, fine aggregates, and palm-nut fiber are all used in the concrete mix. For water, cement, fine and coarse aggregate and palm nut fiber, respectively, the highest flexural strength of 11.40 N/mm^2^ was obtained with a ratio of 0.525:1.0:1.45:1.75:0.6, while the minimum flexural strength of 5.35 N/mm^2^ was obtained with a ratio of 0.6:1.0:2.0:2.8:1.1. The proposed model’s performance was evaluated using statistical methods, which revealed that the model and experimental findings are not significantly different. Additionally, in their research study on the evaluation of crushed recycled ceramics tiles (CRTs), Edidiong et al. [23] aggregated concrete’s mechanical properties using Scheffe’s optimization theory. From the experimental or laboratory test results obtained, the incorporation of CRT as fine aggregates linearly increased the mechanical strength responses as its content in the concrete matrix increases. The formulated Scheffe’s regression model could calculate the cost, compressive strength and slump of the CRT concrete and can be validated using the analysis of variance statistical method at 5% critical value.

The aim of this research was to evaluate green pervious five-component concrete’s flexural strength behavior using Scheffe’s optimization quadratic polynomial model with industrial wastes and their derivatives, namely using sawdust ash (SDA) and quarry dust (QD) as the mineral admixtures [24]. The benefits derived from this experimental investigation seek to ascertain the optimum combination ratio of the five component mixture ingredients constituting of water, cement, quarry dust, coarse aggregates and sawdust ash as well as assess the SDA and QD effects on the response property through morphological and mineralogical assessments of the blended pervious concrete mixture [25]. Scheffe’s second-order polynomial model is thus utilized for the optimization of the mechanical property of pervious concrete consisting of quarry dust with cementitious content partially replaced with sawdust ash (SDA) as a supplementary cementitious material (SCM) in this research study. This helps in the prediction of the concrete’s response in terms of a real valued function for applicability purposes to produce concrete for the desired environmental and design conditions. The utilization of solid waste derivatives in construction works is a major research area in concrete development studies for the application of pozzolanic behavior as well as to reduce the cost and challenges associated with greenhouse gasses emissions which degrade our environment [26,27].

## 2. Materials and Methodology

### 2.1. Materials

#### 2.1.1. Portland Cement

In this investigation, 30% normal consistency Dangote cement (42.5 grade) was employed, which met the requirements for cement class (CEM II) as defined by Nigerian Industrial Standard (NIS) 444–1 specifications [28] in terms of composition and conformance criteria.

#### 2.1.2. Water

Water is a key component that impacts the mechanical and rheological qualities of concrete. For this experiment, clean drinkable water was used, and it met the ASTM C1602-12 water requirement for use in concrete mixtures [29].

#### 2.1.3. Quarry Dust (QD)

QD is a by-product of the crushing or breaking down of granite stones into various sizes of coarse aggregates. The QD utilized in this study came from a quarry in Nigeria’s Cross River State. The required number of QD samples was obtained, which were then sundried, stored and prepared for testing in accordance with ASTM C 618 [30].

#### 2.1.4. Coarse Aggregates

In the experimental examination, crushed granite with a size of 20 mm, which was downgraded to 4.75 mm and graded accordingly, was obtained from a local stone market and conformed to BS EN12620 [31].

#### 2.1.5. Sawdust Ash (SDA)

Timber Wood Workshop in Owerri, Imo State, provided the sawdust. The industrial residue was then burned using a regulated incineration system, yielding ash samples that were sieved using a 150 m sieve size and processed for laboratory testing in line with BS 8615-1 (2019) and ASTM C618. [30,32].

### 2.2. Methods

#### Experimental Investigation and Setup

**Figure 1:**
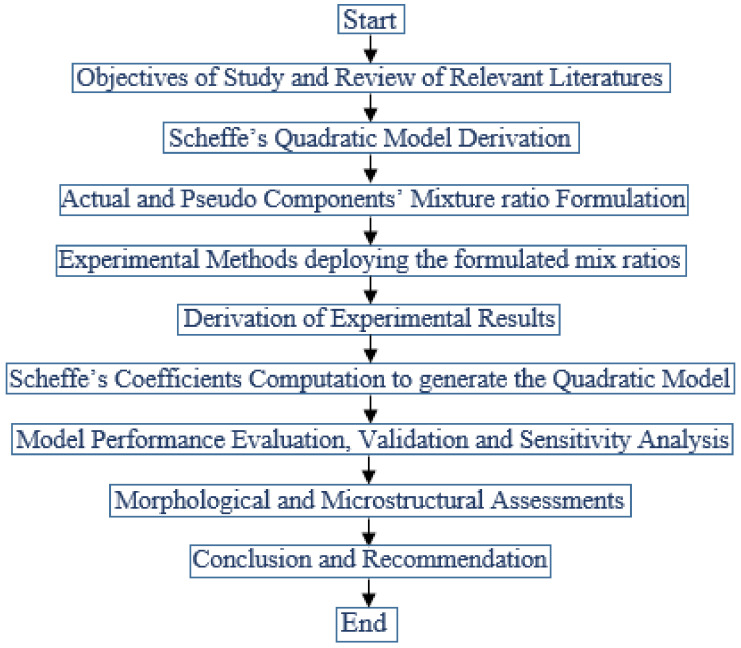
Scheffe’s model development flow chart methodology (source: Attah et al. [19]).

This experimental program comprises a pervious concrete mixture with the five components of cement, water, quarry dust, fine aggregates and sawdust ash in the matrix. Using Scheffe’s simplex lattice statistical approach, the formulation of the mixture ingredients’ combinations for the experiments was determined within the designed factor space using the mathematical relationship between the actual and pseudo-components [33]. The formulated mixture ingredients ratios were converted into an effective mass using the density–mass–volume relation and taking the standard concrete density of 2400 kg/m^3^ and the beam mold volume of 0.004 m^3^. Before compaction and installation in the mold for mechanical strength tests, the concrete mixture materials were thoroughly mixed with water to achieve a homogeneous mixture. On the fresh concrete, the tests were conducted to determine the setting time and workability features. The concrete samples were immersed in a curing tank for 28 days at room temperature after 24 h [34,35]. The flow chart in Figure 1 depicts the process of Scheffe’s model creation, which was adapted for this study.

### 2.3. Mixture Components Formulation

#### 2.3.1. Design of Experiments

The design of experiments constitutes a systematic assessment of the factor levels or component variable effects of the mixture in a simultaneous manner on the target response function, which is achieved using response surface methodology [36]. The deployment of this essential tool in laboratory experiments research helps to yield the minimization of cost and time resources by the generation of a maximum quantity of information for limited laboratory test trials. It also helps in the determination of feasible experimental points where the desired responses should be evaluated so as to establish relationships between factor levels known as the independent variables and the response parameters [37,38]. Mixtures are very essential to industrialization and infrastructural development works. Any two or more components can combine together to produce a mixture; it is the proportions rather than the quantities of ingredients in the mixture that influence the response parameters. Henry Scheffe’s pioneering article on experiments with mixture laid a solid foundation for mixture tools and technique development by presenting the simplex lattice designs with their corresponding canonical polynomials [39,40].

#### 2.3.2. Scheffe’s Simplex Lattice Design

Simplex lattice design is a mixture experiment method which presents a general modeling response method for evaluating component ingredient relationships with dependent variables. This form of mixed experiment is mostly used or adapted in situations where the response factor is determined by the proportions of mixture ingredients rather than their total mass. This is typical of concrete material properties with a *q* total number of factor levels (*x_i_*) for *i* = 1, 2, 3, …, *q*; the desired response (*y_i_*) for a *q*-component mixture is presented in Equation (1) [41].

(1) $$y=f\left(x_{1},x_{2},\ldots ,x_{q}\right)$$

To design a mixture experiment, the major boundary conditions indicate that no component in the mixture should possess a negative value as well as sum up to one constraint, as shown in Equations (2) and (3)

(2) $$\sum_{i=1}^{q}x_{i}=1\mathrm{for}\;0\leq x_{i}\leq 1$$

(3) $$i.e.,\;x_{1}+x_{2}+\ldots +x_{q}=1$$

A lattice is defined as a regular pattern or orderly distribution of points in an abstract structure representation. Claringbold [42] was the first to introduce the simplex lattice approach in his research on combined action on allied hormones. The simplex lattice design, on the other hand, was further expanded and generalized by Scheffe [39] for the statistical evaluation of the effects of factor levels on the response function. His work is frequently regarded as a forerunner in the field of simplex lattice mixture design. Scheffe’s simplex lattice patterns are now a popular term for lattice designs. He claimed that every component in the mixture is at the vertex of a regular simplex-lattice with *q*-1 factor space. However, since the sum of mixture ingredients is constrained to unity, then (q−1) of the factor variables can be chosen autonomously; therefore, from Equation (1), we obtain the mathematical expression, as shown in Equation (4) [43].

(4) $$\sum_{i=1}^{q-1}x_{i}-1=x_{q}$$

**Figure 2:**
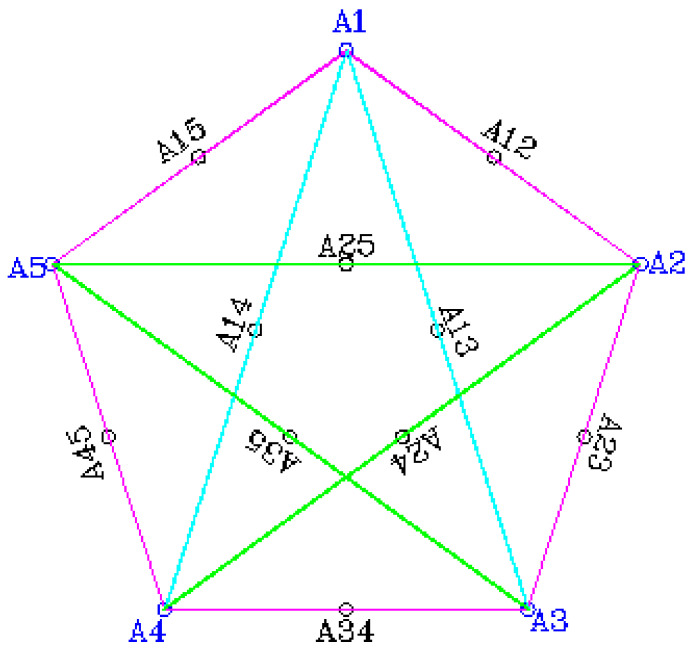
Defined experimental points using Scheffe’s simplex lattice.

Based on the sum of one constraint mathematically expressed in Equations (2) and (3), the well-defined experimental region or factor space obtained by imposing this limitation is the unvarying tetrahedron for a (5,2) simplex region, as shown in Figure 2 [44].

The design experimental points’ number required for the derivation of an optimum concentration of mixture ingredients, which is also known as number terms for Scheffe’s reduced polynomials, is given as $C_{m}^{q+m}$—where q denotes the ingredients’ number in the mixture and *m* denotes the order of the regression polynomial. These number terms define the required number of regression coefficients of the developed model, as expressed in an expanded form, as shown in Equation (5) [45].

(5) $$N=C_{m}^{q+m-1}=\frac{\left(q+m-1\right)!}{\left(q-1\right)!\left(m\right)!}=\frac{\left(5+2-1\right)!}{\left(5-1\right)!\left(2\right)!}=15$$

The mixture characteristics are described using the mathematical (polynomial) function of the m-order and *q*, which is the mixture components’ number to obtain a (q,m) polynomial of the general form, as shown in Equation (5):

(6) $$Y=b_{0}+\sum b_{i}x_{i}+\sum b_{ij}x_{i}x_{j}+\sum b_{ijk}x_{i}x_{j}x_{k}+\sum b_{i1,i2}\ldots i_{n}x_{i1}x_{i2}x_{im}$$

for (1≤i≤q,1≤i≤j≤q,1≤i≤j≤k≤q) where $b_{i}$ is the pure blend linear mixing segments, and $x_{i}$ = 1 and $x_{j}$ = 0; i≠j≠k; E(y) are the predicted output. $b_{ij}$ denotes the nonlinear quadratic mixing factor between the pairs of mixture ingredients and $b_{ijk}$ signifies the coefficients of cubic nonlinear blending between factor levels whereby their characteristics may either be synergistic or antagonistic blending [46].

#### 2.3.3. Derivation of Scheffe’s Second Order Response Function

In the factorization of Equation (5), the further substitution of boundary conditions (0≤i≤j≤5) transforms into Equation (6).

Y = b_0_ + b_1_X_1_ + b_2_X_2_ + b_3_X_3_ + b_4_X_4_ + b_5_X_5_ + b_11_X_1_^2^ + b_12_X_1_X_2_ + b_13_X_1_X_3_ + b_14_X_1_X_4_ + b_15_X_1_X_5_ + b_22_X_2_^2^ + b_23_X_2_X_3_ + b_24_X_2_X_4_ + b_25_X_2_X_5_ + b_33_X_3_^2^ + b_34_X_3_X_4_ + b_35_X_3_X_5_ + b_44_X_4_^2^ + b_45_X_4_X_5_ + b_55_X_5_^2^ (7)

Through the multiplication of b_0_ by Equation (3), we obtain the mathematical expression shown in Equation (7)

b_0_ = b_0_ (X_1_ + X_2_ + X_3_ + X_4_ + X_5_) (8)

Multiplying Equation (2) by X_i_ in succession, we derive the relationship in Equation (8)

X_1_^2^ = X_1_(1 − X_2_ − X_3_ − X_4_ − X_5_)

X_2_^2^ = X_2_(1 − X_1_ − X_3_ − X_4_ − X_5_)

X_3_^2^ = X_3_ (1 − X_1_ − X_2_ − X_4_ − X_5_) (9)

X_4_^2^ = X_4_(1 − X_1_ − X_2_ − X_3_ − X_5_)

X_5_^2^ = X_5_(1 − X_1_ − X_2_ − X_3_ − X_4_)

Substituting Equations (7) and (8) into Equation (6), we achieve the polynomial model in second-order form for five mixture components; we obtain an expression shown in Equation (9).

(10) $$E\left(y\right)=X_{1}(b_{0}+b_{1}+b_{11})+X_{2}(b_{0}+b_{2}+b_{22})+X_{3}(b_{0}+b_{3}+b_{33})+X_{4}(b_{0}+b_{4}+b_{44})+X_{5}(b_{0}+b_{5}+b_{55})+X_{1}X_{2}(b_{12}-b_{11}\;-b_{22})+X_{1}X_{3}(b_{13}-b_{11}-b_{33})+X_{1}X_{4}(b_{14}-b_{11}-b_{44})+X_{1}X_{5}(b_{15}-b_{11}-b_{55})+X_{2}X_{3}(b_{23}-b_{22}-b_{33})+X_{2}X_{4}(b_{24}-b_{22}-\;b_{44})+X_{2}X_{5}(b_{25}-b_{11}-b_{55})+X_{3}X_{4}(b_{34}-b_{33}-b_{44})+X_{3}X_{5}(b_{35}-b_{33}-b_{55})+X_{4}X_{5}(b_{45}-b_{44}-b_{55})$$

We denote the mathematical relationship for the derivation of Scheffe’s regression coefficients, as shown in Equations (10) and (11)

*β*_i_ = b_0_ + b_i_ + b_ii_ (11)

*β*_ij_ = b_ij_ − b_ii_ − b_jj_ (12)

Then, substituting these Equations (10) and (11), we arrive at the reduced second-degree polynomial presented in Equation (12):

(13) $$\hat{Y}=\beta_{1}X_{1}+\beta_{2}X_{2}+\beta_{3}X_{3}+\beta_{4}X_{4}+\beta_{5}X_{5}+\beta_{12}X_{1}X_{2}+\beta_{13}X_{1}X_{3}+\beta_{14}X_{1}X_{4}+\beta_{15}X_{1}X_{5}+\beta_{23}X_{2}X_{3}+\beta_{24}X_{2}X_{4}+\beta_{25}X_{2}X_{5}+\;\beta_{34}X_{3}X_{4}+\beta_{35}X_{3}X_{5}+\beta_{45}X_{4}X_{5}$$

The pseudo-components for the mixture design is denoted by X_i_ while the response coefficients of Scheffe’s optimization equation is denoted by *β*_i_. These regression coefficients can be pure or binary blends, expressed as *β*_i_, and the ternary blends or the combination of the mixture components represented as *β*_ij_. The mathematical definition is shown in Equation (13) [47]

(14) $$\beta_{i}=Y_{i}\;\mathrm{and}\;\beta_{\mathrm{ij}}=4Y_{ij}-2Y_{i}-2Y_{j}$$

Thus, the relationships for the derivation of model coefficients are presented in Equation (14):

β_12_ = 4Y_12_ − 2Y_1_ − 2Y_2_, β_13_ = 4Y_13_ − 2Y_1_ − 2Y_3_, β_14_ = 4Y_14_ − 2Y_1_ − 2Y_4_, β_15_ = 4Y_15_ − 2Y_1_ − 2Y_5_,

β_23_ = 4Y_23_ − 2Y_2_ − 2Y_3_, β_24_ = 4Y_24_ − 2Y_2_ − 2Y_4_, β_25_ = 4Y_25_ − 2Y_2_ − 2Y_5_, β_34_ = 4Y_34_ − 2Y_3_ − 2Y_4,_ (15)

β_35_ = 4Y_35_ − 2Y_3_ − 2Y_5,_ β_45_ = 4Y_45_ − 2Y_4_ − 2Y_5_

#### 2.3.4. Actual Components and Pseudo-Components

Pseudo-components are imaginary or coded variables that are used to facilitate design creation and model fitting in limited designs by minimizing the correlation between component boundaries. The transformation of actual components Z_i_ into pseudo-components X_i_ effectively modifies the constrained data-space in Scheffe’s method so that the lower- and upper-limit values of each factor level are 0 and 1, respectively, in the design factor space, and the mathematical relationships with the actual values are presented in Equation (15).

(16) $$\{Z_{i}\}=[A]\times \{X_{i}\}\;$$

Z_i_ denotes the real or actual proportion of ingredients, whilst X_i_ signifies that the pseudo ratios of these two parameters are in vector form, and A is the constant and in matrix form. Matrix A is derived from the five initial actual mix proportion values. These initial trial component fraction mixes generate the matrix A of q×q dimension [48,49].

### 2.4. Mix Ratio Development

Expert judgment, experience, economical aspects and related literature works were consulted in the generation of the initial trial mixes to take off the computation of the interaction points using Equation (18). This generation of the initial mix was carried out separately for the flexural and compressive strength evaluation, respectively [50,51].

#### Mixture Formulation Computation

The initial mix ratios were Z_1_ [0.435:0.95:0.1:1.55:0.05], Z_2_ [0.45:0.9:0.13:1.95:0.1], Z_3_ [0.5:0.85:0.19:2.85:0.15], Z_4_ [0.55:0.8:0.25:3.55:0.2] and Z_5_ [0.6:0.75:0.3:4.1:0.25].

The equivalent pseudo-component values which are in binary form, indicating the pure blend in the mix configuration, are X_1_ [1:0:0:0:0], X_2_ [0:1:0:0:0], X_3_ [0:0:1:0:0], X_4_ [0:0:0:1:0] and X_5_ [0:0:0:0:1].

The substitution of X_i_ and Z_i_ into Equation (15) helps calculate the pseudo-components from the resulting actual mixture components.

X_1_ = water–cement ratio fraction; X_2_ = Portland cement fraction; X_3_ = quarry dust fraction; X_4_ = coarse aggregate fraction; and X_5_ = sawdust ash fraction

The equation is transformed into the matrix notation for the computation of the experimental mixture proportions [48].

$$\left(\begin{array}{c} Z_{1}\\ Z_{2}\\ \begin{array}{c} Z_{3}\\ \begin{array}{c} Z_{4}\\ Z_{5} \end{array} \end{array} \end{array}\right)=\left(\begin{array}{c} a_{11}\\ a_{21}\\ \begin{array}{c} a_{31}\\ \begin{array}{c} a_{41}\\ a_{51} \end{array} \end{array} \end{array}\;\;\;\;\;\;\;\;\begin{array}{c} a_{12}\\ a_{22}\\ \begin{array}{c} a_{32}\\ \begin{array}{c} a_{42}\\ a_{52} \end{array} \end{array} \end{array}\;\;\;\;\;\;\;\;\begin{array}{c} a_{13}\\ a_{23}\\ \begin{array}{c} a_{33}\\ \begin{array}{c} a_{43}\\ a_{53} \end{array} \end{array} \end{array}\;\;\;\;\;\;\;\;\;\;\begin{array}{c} a_{14}\\ a_{24}\\ \begin{array}{c} a_{34}\\ \begin{array}{c} a_{44}\\ a_{54} \end{array} \end{array} \end{array}\;\;\;\;\;\;\;\;\;\;\begin{array}{c} a_{15}\\ a_{25}\\ \begin{array}{c} a_{35}\\ \begin{array}{c} a_{45}\\ a_{55} \end{array} \end{array} \end{array}\right)\;\left(\begin{array}{c} X_{1}\\ X_{2}\\ \begin{array}{c} X_{3}\\ \begin{array}{c} X_{4}\\ X_{5} \end{array} \end{array} \end{array}\right)$$

For the first run

$$\left(\begin{array}{c} 0.435\\ 0.95\\ \begin{array}{c} 0.1\\ \begin{array}{c} 1.55\\ 0.05 \end{array} \end{array} \end{array}\right)=\left(\begin{array}{c} a_{11}\\ a_{21}\\ \begin{array}{c} a_{31}\\ \begin{array}{c} a_{41}\\ a_{51} \end{array} \end{array} \end{array}\;\;\;\;\;\;\;\;\begin{array}{c} a_{12}\\ a_{22}\\ \begin{array}{c} a_{32}\\ \begin{array}{c} a_{42}\\ a_{52} \end{array} \end{array} \end{array}\;\;\;\;\;\;\;\;\begin{array}{c} a_{13}\\ a_{23}\\ \begin{array}{c} a_{33}\\ \begin{array}{c} a_{43}\\ a_{53} \end{array} \end{array} \end{array}\;\;\;\;\;\;\;\;\;\;\begin{array}{c} a_{14}\\ a_{24}\\ \begin{array}{c} a_{34}\\ \begin{array}{c} a_{44}\\ a_{54} \end{array} \end{array} \end{array}\;\;\;\;\;\;\;\;\;\;\begin{array}{c} a_{15}\\ a_{25}\\ \begin{array}{c} a_{35}\\ \begin{array}{c} a_{45}\\ a_{55} \end{array} \end{array} \end{array}\right)\;\left(\begin{array}{c} 1\\ 0\\ \begin{array}{c} 0\\ \begin{array}{c} 0\\ 0 \end{array} \end{array} \end{array}\right)$$

$$a_{11}=\;0.435,\;a_{21}=\;0.95,\;a_{31}=\;0.1,\;a_{41}=\;1.55,\;a_{51}=\;0.05$$

For the second run

$$\left(\begin{array}{c} 0.45\\ 0.9\\ \begin{array}{c} 0.13\\ \begin{array}{c} 1.95\\ 0.1 \end{array} \end{array} \end{array}\right)=\left(\begin{array}{c} a_{11}\\ a_{21}\\ \begin{array}{c} a_{31}\\ \begin{array}{c} a_{41}\\ a_{51} \end{array} \end{array} \end{array}\;\;\;\;\;\;\;\;\begin{array}{c} a_{12}\\ a_{22}\\ \begin{array}{c} a_{32}\\ \begin{array}{c} a_{42}\\ a_{52} \end{array} \end{array} \end{array}\;\;\;\;\;\;\;\;\begin{array}{c} a_{13}\\ a_{23}\\ \begin{array}{c} a_{33}\\ \begin{array}{c} a_{43}\\ a_{53} \end{array} \end{array} \end{array}\;\;\;\;\;\;\;\;\;\;\begin{array}{c} a_{14}\\ a_{24}\\ \begin{array}{c} a_{34}\\ \begin{array}{c} a_{44}\\ a_{54} \end{array} \end{array} \end{array}\;\;\;\;\;\;\;\;\;\;\begin{array}{c} a_{15}\\ a_{25}\\ \begin{array}{c} a_{35}\\ \begin{array}{c} a_{45}\\ a_{55} \end{array} \end{array} \end{array}\right)\;\left(\begin{array}{c} 0\\ 1\\ \begin{array}{c} 0\\ \begin{array}{c} 0\\ 0 \end{array} \end{array} \end{array}\right)$$

$$a_{12}=\;0.45,\;a_{22}=\;0.9,\;a_{32}=\;0.13,\;a_{42}=\;1.95,\;a_{52}=\;0.1$$

For the third run

$$\left(\begin{array}{c} 0.5\\ 0.85\\ \begin{array}{c} 0.19\\ \begin{array}{c} 2.85\\ 0.15 \end{array} \end{array} \end{array}\right)=\left(\begin{array}{c} a_{11}\\ a_{21}\\ \begin{array}{c} a_{31}\\ \begin{array}{c} a_{41}\\ a_{51} \end{array} \end{array} \end{array}\;\;\;\;\;\;\;\;\begin{array}{c} a_{12}\\ a_{22}\\ \begin{array}{c} a_{32}\\ \begin{array}{c} a_{42}\\ a_{52} \end{array} \end{array} \end{array}\;\;\;\;\;\;\;\;\begin{array}{c} a_{13}\\ a_{23}\\ \begin{array}{c} a_{33}\\ \begin{array}{c} a_{43}\\ a_{53} \end{array} \end{array} \end{array}\;\;\;\;\;\;\;\;\;\;\begin{array}{c} a_{14}\\ a_{24}\\ \begin{array}{c} a_{34}\\ \begin{array}{c} a_{44}\\ a_{54} \end{array} \end{array} \end{array}\;\;\;\;\;\;\;\;\;\;\begin{array}{c} a_{15}\\ a_{25}\\ \begin{array}{c} a_{35}\\ \begin{array}{c} a_{45}\\ a_{55} \end{array} \end{array} \end{array}\right)\;\left(\begin{array}{c} 0\\ 0\\ \begin{array}{c} 1\\ \begin{array}{c} 0\\ 0 \end{array} \end{array} \end{array}\right)$$

$$a_{13}=\;0.5,\;a_{23}\;=\;0.85,\;a_{33}\;=\;0.19,\;a_{43}\;=\;2.85,\;a_{53}\;=\;0.15$$

For the fourth run

$$\left(\begin{array}{c} 0.55\\ 0.8\\ \begin{array}{c} 0.25\\ \begin{array}{c} 3.55\\ 0.2 \end{array} \end{array} \end{array}\right)=\left(\begin{array}{c} a_{11}\\ a_{21}\\ \begin{array}{c} a_{31}\\ \begin{array}{c} a_{41}\\ a_{51} \end{array} \end{array} \end{array}\;\;\;\;\;\;\;\;\begin{array}{c} a_{12}\\ a_{22}\\ \begin{array}{c} a_{32}\\ \begin{array}{c} a_{42}\\ a_{52} \end{array} \end{array} \end{array}\;\;\;\;\;\;\;\;\begin{array}{c} a_{13}\\ a_{23}\\ \begin{array}{c} a_{33}\\ \begin{array}{c} a_{43}\\ a_{53} \end{array} \end{array} \end{array}\;\;\;\;\;\;\;\;\;\;\begin{array}{c} a_{14}\\ a_{24}\\ \begin{array}{c} a_{34}\\ \begin{array}{c} a_{44}\\ a_{54} \end{array} \end{array} \end{array}\;\;\;\;\;\;\;\;\;\;\begin{array}{c} a_{15}\\ a_{25}\\ \begin{array}{c} a_{35}\\ \begin{array}{c} a_{45}\\ a_{55} \end{array} \end{array} \end{array}\right)\;\left(\begin{array}{c} 0\\ 0\\ \begin{array}{c} 0\\ \begin{array}{c} 1\\ 0 \end{array} \end{array} \end{array}\right)$$

$$a_{14}=\;0.55,\;a_{24}\;=\;0.8,\;a_{34}=\;0.25,\;a_{44}\;=\;3.55,\;a_{54}\;=\;0.2$$

For the fifth run

$$\left(\begin{array}{c} 0.6\\ 0.75\\ \begin{array}{c} 0.3\\ \begin{array}{c} 4.1\\ 0.25 \end{array} \end{array} \end{array}\right)=\left(\begin{array}{c} a_{11}\\ a_{21}\\ \begin{array}{c} a_{31}\\ \begin{array}{c} a_{41}\\ a_{51} \end{array} \end{array} \end{array}\;\;\;\;\;\;\;\;\begin{array}{c} a_{12}\\ a_{22}\\ \begin{array}{c} a_{32}\\ \begin{array}{c} a_{42}\\ a_{52} \end{array} \end{array} \end{array}\;\;\;\;\;\;\;\;\begin{array}{c} a_{13}\\ a_{23}\\ \begin{array}{c} a_{33}\\ \begin{array}{c} a_{43}\\ a_{53} \end{array} \end{array} \end{array}\;\;\;\;\;\;\;\;\;\;\begin{array}{c} a_{14}\\ a_{24}\\ \begin{array}{c} a_{34}\\ \begin{array}{c} a_{44}\\ a_{54} \end{array} \end{array} \end{array}\;\;\;\;\;\;\;\;\;\;\begin{array}{c} a_{15}\\ a_{25}\\ \begin{array}{c} a_{35}\\ \begin{array}{c} a_{45}\\ a_{55} \end{array} \end{array} \end{array}\right)\;\left(\begin{array}{c} 0\\ 0\\ \begin{array}{c} 0\\ \begin{array}{c} 0\\ 1 \end{array} \end{array} \end{array}\right)$$

$$a_{15}\;=\;0.6,\;a_{25}\;=0.75,\;a_{35}=\;0.3,\;a_{45}\;=\;4.1,\;a_{55}\;=\;0.25$$

Substituting the values of the constants, we have [A] matrix

$$\left(\begin{array}{c} 0.435\\ 0.95\\ \begin{array}{c} 0.10\\ \begin{array}{c} 1.55\\ 0.05 \end{array} \end{array} \end{array}\;\;\;\;\;\;\;\;\begin{array}{c} 0.45\\ 0.9\\ \begin{array}{c} 0.13\\ \begin{array}{c} 1.95\\ 0.1 \end{array} \end{array} \end{array}\;\;\;\;\;\;\;\;\begin{array}{c} 0.5\\ 0.85\\ \begin{array}{c} 0.19\\ \begin{array}{c} 2.85\\ 0.15 \end{array} \end{array} \end{array}\;\;\;\;\;\;\;\;\;\;\begin{array}{c} 0.55\\ 0.8\\ \begin{array}{c} 0.25\\ \begin{array}{c} 3.55\\ 0.2 \end{array} \end{array} \end{array}\;\;\;\;\;\;\;\;\;\;\begin{array}{c} 0.6\\ 0.75\\ \begin{array}{c} 0.3\\ \begin{array}{c} 4.1\\ 0.25 \end{array} \end{array} \end{array}\right)$$

The points at the five vertices of the simplex factor space make up the first five points, and the remaining ten points located inside of the simplex, which are the interaction points, are calculated by substituting Equation (15) as follows

Therefore, for A_12_

$$\left(\begin{array}{c} Z_{1}\\ Z_{2}\\ \begin{array}{c} Z_{3}\\ \begin{array}{c} Z_{4}\\ Z_{5} \end{array} \end{array} \end{array}\right)=\left(\begin{array}{c} 0.435\\ 0.95\\ \begin{array}{c} 0.10\\ \begin{array}{c} 1.55\\ 0.05 \end{array} \end{array} \end{array}\;\;\;\;\;\;\;\;\begin{array}{c} 0.45\\ 0.9\\ \begin{array}{c} 0.13\\ \begin{array}{c} 1.95\\ 0.1 \end{array} \end{array} \end{array}\;\;\;\;\;\;\;\;\begin{array}{c} 0.5\\ 0.85\\ \begin{array}{c} 0.19\\ \begin{array}{c} 2.85\\ 0.15 \end{array} \end{array} \end{array}\;\;\;\;\;\;\;\;\;\;\begin{array}{c} 0.55\\ 0.8\\ \begin{array}{c} 0.25\\ \begin{array}{c} 3.55\\ 0.2 \end{array} \end{array} \end{array}\;\;\;\;\;\;\;\;\;\;\begin{array}{c} 0.6\\ 0.75\\ \begin{array}{c} 0.3\\ \begin{array}{c} 4.1\\ 0.25 \end{array} \end{array} \end{array}\right)*\;\left(\begin{array}{c} 0.5\\ 0.5\\ \begin{array}{c} 0\\ \begin{array}{c} 0\\ 0 \end{array} \end{array} \end{array}\right)=\left(\begin{array}{c} 0.4425\\ 0.925\\ \begin{array}{c} 0.115\\ \begin{array}{c} 1.75\\ 0.075 \end{array} \end{array} \end{array}\right)$$

For A_13_

$$\left(\begin{array}{c} Z_{1}\\ Z_{2}\\ \begin{array}{c} Z_{3}\\ \begin{array}{c} Z_{4}\\ Z_{5} \end{array} \end{array} \end{array}\right)=\left(\begin{array}{c} 0.435\\ 0.95\\ \begin{array}{c} 0.10\\ \begin{array}{c} 1.55\\ 0.05 \end{array} \end{array} \end{array}\;\;\;\;\;\;\;\;\begin{array}{c} 0.45\\ 0.9\\ \begin{array}{c} 0.13\\ \begin{array}{c} 1.95\\ 0.1 \end{array} \end{array} \end{array}\;\;\;\;\;\;\;\;\begin{array}{c} 0.5\\ 0.85\\ \begin{array}{c} 0.19\\ \begin{array}{c} 2.85\\ 0.15 \end{array} \end{array} \end{array}\;\;\;\;\;\;\;\;\;\;\begin{array}{c} 0.55\\ 0.8\\ \begin{array}{c} 0.25\\ \begin{array}{c} 3.55\\ 0.2 \end{array} \end{array} \end{array}\;\;\;\;\;\;\;\;\;\;\begin{array}{c} 0.6\\ 0.75\\ \begin{array}{c} 0.3\\ \begin{array}{c} 4.1\\ 0.25 \end{array} \end{array} \end{array}\right)*\;\left(\begin{array}{c} 0.5\\ 0\\ \begin{array}{c} 0.5\\ \begin{array}{c} 0\\ 0 \end{array} \end{array} \end{array}\right)=\left(\begin{array}{c} 0.4675\\ 0.9\\ \begin{array}{c} 0.145\\ \begin{array}{c} 2.2\\ 0.1 \end{array} \end{array} \end{array}\right)$$

For A_14_

$$\left(\begin{array}{c} Z_{1}\\ Z_{2}\\ \begin{array}{c} Z_{3}\\ \begin{array}{c} Z_{4}\\ Z_{5} \end{array} \end{array} \end{array}\right)=\left(\begin{array}{c} 0.435\\ 0.95\\ \begin{array}{c} 0.10\\ \begin{array}{c} 1.55\\ 0.05 \end{array} \end{array} \end{array}\;\;\;\;\;\;\;\;\begin{array}{c} 0.45\\ 0.9\\ \begin{array}{c} 0.13\\ \begin{array}{c} 1.95\\ 0.1 \end{array} \end{array} \end{array}\;\;\;\;\;\;\;\;\begin{array}{c} 0.5\\ 0.85\\ \begin{array}{c} 0.19\\ \begin{array}{c} 2.85\\ 0.15 \end{array} \end{array} \end{array}\;\;\;\;\;\;\;\;\;\;\begin{array}{c} 0.55\\ 0.8\\ \begin{array}{c} 0.25\\ \begin{array}{c} 3.55\\ 0.2 \end{array} \end{array} \end{array}\;\;\;\;\;\;\;\;\;\;\begin{array}{c} 0.6\\ 0.75\\ \begin{array}{c} 0.3\\ \begin{array}{c} 4.1\\ 0.25 \end{array} \end{array} \end{array}\right)*\;\left(\begin{array}{c} 0.5\\ 0\\ \begin{array}{c} 0\\ \begin{array}{c} 0.5\\ 0 \end{array} \end{array} \end{array}\right)=\left(\begin{array}{c} 0.4925\\ 0.875\\ \begin{array}{c} 0.175\\ \begin{array}{c} 2.55\\ 0.125 \end{array} \end{array} \end{array}\right)$$

For A_15_

$$\left(\begin{array}{c} Z_{1}\\ Z_{2}\\ \begin{array}{c} Z_{3}\\ \begin{array}{c} Z_{4}\\ Z_{5} \end{array} \end{array} \end{array}\right)=\left(\begin{array}{c} 0.435\\ 0.95\\ \begin{array}{c} 0.10\\ \begin{array}{c} 1.55\\ 0.05 \end{array} \end{array} \end{array}\;\;\;\;\;\;\;\;\begin{array}{c} 0.45\\ 0.9\\ \begin{array}{c} 0.13\\ \begin{array}{c} 1.95\\ 0.1 \end{array} \end{array} \end{array}\;\;\;\;\;\;\;\;\begin{array}{c} 0.5\\ 0.85\\ \begin{array}{c} 0.19\\ \begin{array}{c} 2.85\\ 0.15 \end{array} \end{array} \end{array}\;\;\;\;\;\;\;\;\;\;\begin{array}{c} 0.55\\ 0.8\\ \begin{array}{c} 0.25\\ \begin{array}{c} 3.55\\ 0.2 \end{array} \end{array} \end{array}\;\;\;\;\;\;\;\;\;\;\begin{array}{c} 0.6\\ 0.75\\ \begin{array}{c} 0.3\\ \begin{array}{c} 4.1\\ 0.25 \end{array} \end{array} \end{array}\right)*\;\left(\begin{array}{c} 0.5\\ 0\\ \begin{array}{c} 0\\ \begin{array}{c} 0\\ 0.5 \end{array} \end{array} \end{array}\right)=\left(\begin{array}{c} 0.5175\\ 0.85\\ \begin{array}{c} 0.2\\ \begin{array}{c} 2.825\\ 0.5 \end{array} \end{array} \end{array}\right)$$

For A_23_

$$\left(\begin{array}{c} Z_{1}\\ Z_{2}\\ \begin{array}{c} Z_{3}\\ \begin{array}{c} Z_{4}\\ Z_{5} \end{array} \end{array} \end{array}\right)=\left(\begin{array}{c} 0.435\\ 0.95\\ \begin{array}{c} 0.10\\ \begin{array}{c} 1.55\\ 0.05 \end{array} \end{array} \end{array}\;\;\;\;\;\;\;\;\begin{array}{c} 0.45\\ 0.9\\ \begin{array}{c} 0.13\\ \begin{array}{c} 1.95\\ 0.1 \end{array} \end{array} \end{array}\;\;\;\;\;\;\;\;\begin{array}{c} 0.5\\ 0.85\\ \begin{array}{c} 0.19\\ \begin{array}{c} 2.85\\ 0.15 \end{array} \end{array} \end{array}\;\;\;\;\;\;\;\;\;\;\begin{array}{c} 0.55\\ 0.8\\ \begin{array}{c} 0.25\\ \begin{array}{c} 3.55\\ 0.2 \end{array} \end{array} \end{array}\;\;\;\;\;\;\;\;\;\;\begin{array}{c} 0.6\\ 0.75\\ \begin{array}{c} 0.3\\ \begin{array}{c} 4.1\\ 0.25 \end{array} \end{array} \end{array}\right)*\;\left(\begin{array}{c} 0\\ 0.5\\ \begin{array}{c} 0.5\\ \begin{array}{c} 0\\ 0 \end{array} \end{array} \end{array}\right)=\left(\begin{array}{c} 0.475\\ 0.875\\ \begin{array}{c} 0.16\\ \begin{array}{c} 2.4\\ 0.125 \end{array} \end{array} \end{array}\right)$$

For A_24_

$$\left(\begin{array}{c} Z_{1}\\ Z_{2}\\ \begin{array}{c} Z_{3}\\ \begin{array}{c} Z_{4}\\ Z_{5} \end{array} \end{array} \end{array}\right)=\left(\begin{array}{c} 0.435\\ 0.95\\ \begin{array}{c} 0.10\\ \begin{array}{c} 1.55\\ 0.05 \end{array} \end{array} \end{array}\;\;\;\;\;\;\;\;\begin{array}{c} 0.45\\ 0.9\\ \begin{array}{c} 0.13\\ \begin{array}{c} 1.95\\ 0.1 \end{array} \end{array} \end{array}\;\;\;\;\;\;\;\;\begin{array}{c} 0.5\\ 0.85\\ \begin{array}{c} 0.19\\ \begin{array}{c} 2.85\\ 0.15 \end{array} \end{array} \end{array}\;\;\;\;\;\;\;\;\;\;\begin{array}{c} 0.55\\ 0.8\\ \begin{array}{c} 0.25\\ \begin{array}{c} 3.55\\ 0.2 \end{array} \end{array} \end{array}\;\;\;\;\;\;\;\;\;\;\begin{array}{c} 0.6\\ 0.75\\ \begin{array}{c} 0.3\\ \begin{array}{c} 4.1\\ 0.25 \end{array} \end{array} \end{array}\right)*\;\left(\begin{array}{c} 0\\ 0.5\\ \begin{array}{c} 0\\ \begin{array}{c} 0.5\\ 0 \end{array} \end{array} \end{array}\right)=\left(\begin{array}{c} 0.5\\ 0.85\\ \begin{array}{c} 0.19\\ \begin{array}{c} 2.75\\ 0.15 \end{array} \end{array} \end{array}\right)$$

For A_25_

$$\left(\begin{array}{c} Z_{1}\\ Z_{2}\\ \begin{array}{c} Z_{3}\\ \begin{array}{c} Z_{4}\\ Z_{5} \end{array} \end{array} \end{array}\right)=\left(\begin{array}{c} 0.435\\ 0.95\\ \begin{array}{c} 0.10\\ \begin{array}{c} 1.55\\ 0.05 \end{array} \end{array} \end{array}\;\;\;\;\;\;\;\;\begin{array}{c} 0.45\\ 0.9\\ \begin{array}{c} 0.13\\ \begin{array}{c} 1.95\\ 0.1 \end{array} \end{array} \end{array}\;\;\;\;\;\;\;\;\begin{array}{c} 0.5\\ 0.85\\ \begin{array}{c} 0.19\\ \begin{array}{c} 2.85\\ 0.15 \end{array} \end{array} \end{array}\;\;\;\;\;\;\;\;\;\;\begin{array}{c} 0.55\\ 0.8\\ \begin{array}{c} 0.25\\ \begin{array}{c} 3.55\\ 0.2 \end{array} \end{array} \end{array}\;\;\;\;\;\;\;\;\;\;\begin{array}{c} 0.6\\ 0.75\\ \begin{array}{c} 0.3\\ \begin{array}{c} 4.1\\ 0.25 \end{array} \end{array} \end{array}\right)*\;\left(\begin{array}{c} 0\\ 0.5\\ \begin{array}{c} 0\\ \begin{array}{c} 0\\ 0.5 \end{array} \end{array} \end{array}\right)=\left(\begin{array}{c} 0.525\\ 0.825\\ \begin{array}{c} 0.215\\ \begin{array}{c} 3.025\\ 0.175 \end{array} \end{array} \end{array}\right)$$

For A_34_

$$\left(\begin{array}{c} Z_{1}\\ Z_{2}\\ \begin{array}{c} Z_{3}\\ \begin{array}{c} Z_{4}\\ Z_{5} \end{array} \end{array} \end{array}\right)=\left(\begin{array}{c} 0.435\\ 0.95\\ \begin{array}{c} 0.10\\ \begin{array}{c} 1.55\\ 0.05 \end{array} \end{array} \end{array}\;\;\;\;\;\;\;\;\begin{array}{c} 0.45\\ 0.9\\ \begin{array}{c} 0.13\\ \begin{array}{c} 1.95\\ 0.1 \end{array} \end{array} \end{array}\;\;\;\;\;\;\;\;\begin{array}{c} 0.5\\ 0.85\\ \begin{array}{c} 0.19\\ \begin{array}{c} 2.85\\ 0.15 \end{array} \end{array} \end{array}\;\;\;\;\;\;\;\;\;\;\begin{array}{c} 0.55\\ 0.8\\ \begin{array}{c} 0.25\\ \begin{array}{c} 3.55\\ 0.2 \end{array} \end{array} \end{array}\;\;\;\;\;\;\;\;\;\;\begin{array}{c} 0.6\\ 0.75\\ \begin{array}{c} 0.3\\ \begin{array}{c} 4.1\\ 0.25 \end{array} \end{array} \end{array}\right)*\;\left(\begin{array}{c} 0\\ 0\\ \begin{array}{c} 0.5\\ \begin{array}{c} 0.5\\ 0 \end{array} \end{array} \end{array}\right)=\left(\begin{array}{c} 0.525\\ 0.825\\ \begin{array}{c} 0.22\\ \begin{array}{c} 3.2\\ 0.178 \end{array} \end{array} \end{array}\right)$$

For A_35_

$$\left(\begin{array}{c} Z_{1}\\ Z_{2}\\ \begin{array}{c} Z_{3}\\ \begin{array}{c} Z_{4}\\ Z_{5} \end{array} \end{array} \end{array}\right)=\left(\begin{array}{c} 0.435\\ 0.95\\ \begin{array}{c} 0.10\\ \begin{array}{c} 1.55\\ 0.05 \end{array} \end{array} \end{array}\;\;\;\;\;\;\;\;\begin{array}{c} 0.45\\ 0.9\\ \begin{array}{c} 0.13\\ \begin{array}{c} 1.95\\ 0.1 \end{array} \end{array} \end{array}\;\;\;\;\;\;\;\;\begin{array}{c} 0.5\\ 0.85\\ \begin{array}{c} 0.19\\ \begin{array}{c} 2.85\\ 0.15 \end{array} \end{array} \end{array}\;\;\;\;\;\;\;\;\;\;\begin{array}{c} 0.55\\ 0.8\\ \begin{array}{c} 0.25\\ \begin{array}{c} 3.55\\ 0.2 \end{array} \end{array} \end{array}\;\;\;\;\;\;\;\;\;\;\begin{array}{c} 0.6\\ 0.75\\ \begin{array}{c} 0.3\\ \begin{array}{c} 4.1\\ 0.25 \end{array} \end{array} \end{array}\right)*\;\left(\begin{array}{c} 0\\ 0\\ \begin{array}{c} 0.5\\ \begin{array}{c} 0\\ 0.5 \end{array} \end{array} \end{array}\right)=\left(\begin{array}{c} 0.55\\ 0.8\\ \begin{array}{c} 0.245\\ \begin{array}{c} 3.475\\ 0.2 \end{array} \end{array} \end{array}\right)$$

For A_45_

$$\left(\begin{array}{c} Z_{1}\\ Z_{2}\\ \begin{array}{c} Z_{3}\\ \begin{array}{c} Z_{4}\\ Z_{5} \end{array} \end{array} \end{array}\right)=\left(\begin{array}{c} 0.435\\ 0.95\\ \begin{array}{c} 0.10\\ \begin{array}{c} 1.55\\ 0.05 \end{array} \end{array} \end{array}\;\;\;\;\;\;\;\;\begin{array}{c} 0.45\\ 0.9\\ \begin{array}{c} 0.13\\ \begin{array}{c} 1.95\\ 0.1 \end{array} \end{array} \end{array}\;\;\;\;\;\;\;\;\begin{array}{c} 0.5\\ 0.85\\ \begin{array}{c} 0.19\\ \begin{array}{c} 2.85\\ 0.15 \end{array} \end{array} \end{array}\;\;\;\;\;\;\;\;\;\;\begin{array}{c} 0.55\\ 0.8\\ \begin{array}{c} 0.25\\ \begin{array}{c} 3.55\\ 0.2 \end{array} \end{array} \end{array}\;\;\;\;\;\;\;\;\;\;\begin{array}{c} 0.6\\ 0.75\\ \begin{array}{c} 0.3\\ \begin{array}{c} 4.1\\ 0.25 \end{array} \end{array} \end{array}\right)*\;\left(\begin{array}{c} 0\\ 0\\ \begin{array}{c} 0.5\\ \begin{array}{c} 0\\ 0.5 \end{array} \end{array} \end{array}\right)=\left(\begin{array}{c} 0.575\\ 0.775\\ \begin{array}{c} 0.275\\ \begin{array}{c} 3.825\\ 0.225 \end{array} \end{array} \end{array}\right)$$

The matrix table for the mixture proportion formulation is shown in Table 1.

Similarly, applying the actual and pseudo mathematical relationships presented in Equation (15), the next fifteen control points are calculated and designed for the authentication of the generated Scheffe’s regression model.

- For second-order control points

For C_1_

$$\left(\begin{array}{c} Z_{1}\\ Z_{2}\\ \begin{array}{c} Z_{3}\\ \begin{array}{c} Z_{4}\\ Z_{5} \end{array} \end{array} \end{array}\right)=\left(\begin{array}{c} 0.435\\ 0.95\\ \begin{array}{c} 0.10\\ \begin{array}{c} 1.55\\ 0.05 \end{array} \end{array} \end{array}\;\;\;\;\;\;\;\;\begin{array}{c} 0.45\\ 0.9\\ \begin{array}{c} 0.13\\ \begin{array}{c} 1.95\\ 0.1 \end{array} \end{array} \end{array}\;\;\;\;\;\;\;\;\begin{array}{c} 0.5\\ 0.85\\ \begin{array}{c} 0.19\\ \begin{array}{c} 2.85\\ 0.15 \end{array} \end{array} \end{array}\;\;\;\;\;\;\;\;\;\;\begin{array}{c} 0.55\\ 0.8\\ \begin{array}{c} 0.25\\ \begin{array}{c} 3.55\\ 0.2 \end{array} \end{array} \end{array}\;\;\;\;\;\;\;\;\;\;\begin{array}{c} 0.6\\ 0.75\\ \begin{array}{c} 0.3\\ \begin{array}{c} 4.1\\ 0.25 \end{array} \end{array} \end{array}\right)*\;\left(\begin{array}{c} 0.25\\ 0.25\\ \begin{array}{c} 0.25\\ \begin{array}{c} 0.25\\ 0 \end{array} \end{array} \end{array}\right)=\left(\begin{array}{c} 0.48375\\ 0.875\\ \begin{array}{c} 0.1675\\ \begin{array}{c} 2.475\\ 0.125 \end{array} \end{array} \end{array}\right)$$

For C_2_

$$\left(\begin{array}{c} Z_{1}\\ Z_{2}\\ \begin{array}{c} Z_{3}\\ \begin{array}{c} Z_{4}\\ Z_{5} \end{array} \end{array} \end{array}\right)=\left(\begin{array}{c} 0.435\\ 0.95\\ \begin{array}{c} 0.10\\ \begin{array}{c} 1.55\\ 0.05 \end{array} \end{array} \end{array}\;\;\;\;\;\;\;\;\begin{array}{c} 0.45\\ 0.9\\ \begin{array}{c} 0.13\\ \begin{array}{c} 1.95\\ 0.1 \end{array} \end{array} \end{array}\;\;\;\;\;\;\;\;\begin{array}{c} 0.5\\ 0.85\\ \begin{array}{c} 0.19\\ \begin{array}{c} 2.85\\ 0.15 \end{array} \end{array} \end{array}\;\;\;\;\;\;\;\;\;\;\begin{array}{c} 0.55\\ 0.8\\ \begin{array}{c} 0.25\\ \begin{array}{c} 3.55\\ 0.2 \end{array} \end{array} \end{array}\;\;\;\;\;\;\;\;\;\;\begin{array}{c} 0.6\\ 0.75\\ \begin{array}{c} 0.3\\ \begin{array}{c} 4.1\\ 0.25 \end{array} \end{array} \end{array}\right)*\;\left(\begin{array}{c} 0.25\\ 0.25\\ \begin{array}{c} 0.25\\ \begin{array}{c} 0\\ 0.25 \end{array} \end{array} \end{array}\right)=\left(\begin{array}{c} 0.49625\\ 0.8625\\ \begin{array}{c} 0.18\\ \begin{array}{c} 2.6125\\ 0.1375 \end{array} \end{array} \end{array}\right)$$

For C_3_

$$\left(\begin{array}{c} Z_{1}\\ Z_{2}\\ \begin{array}{c} Z_{3}\\ \begin{array}{c} Z_{4}\\ Z_{5} \end{array} \end{array} \end{array}\right)=\left(\begin{array}{c} 0.435\\ 0.95\\ \begin{array}{c} 0.10\\ \begin{array}{c} 1.55\\ 0.05 \end{array} \end{array} \end{array}\;\;\;\;\;\;\;\;\begin{array}{c} 0.45\\ 0.9\\ \begin{array}{c} 0.13\\ \begin{array}{c} 1.95\\ 0.1 \end{array} \end{array} \end{array}\;\;\;\;\;\;\;\;\begin{array}{c} 0.5\\ 0.85\\ \begin{array}{c} 0.19\\ \begin{array}{c} 2.85\\ 0.15 \end{array} \end{array} \end{array}\;\;\;\;\;\;\;\;\;\;\begin{array}{c} 0.55\\ 0.8\\ \begin{array}{c} 0.25\\ \begin{array}{c} 3.55\\ 0.2 \end{array} \end{array} \end{array}\;\;\;\;\;\;\;\;\;\;\begin{array}{c} 0.6\\ 0.75\\ \begin{array}{c} 0.3\\ \begin{array}{c} 4.1\\ 0.25 \end{array} \end{array} \end{array}\right)*\;\left(\begin{array}{c} 0.25\\ 0.25\\ \begin{array}{c} 0\\ \begin{array}{c} 0.25\\ 0.25 \end{array} \end{array} \end{array}\right)=\left(\begin{array}{c} 0.50875\\ 0.85\\ \begin{array}{c} 0.195\\ \begin{array}{c} 2.7875\\ 0.15 \end{array} \end{array} \end{array}\right)$$

For C_4_

$$\left(\begin{array}{c} Z_{1}\\ Z_{2}\\ \begin{array}{c} Z_{3}\\ \begin{array}{c} Z_{4}\\ Z_{5} \end{array} \end{array} \end{array}\right)=\left(\begin{array}{c} 0.435\\ 0.95\\ \begin{array}{c} 0.10\\ \begin{array}{c} 1.55\\ 0.05 \end{array} \end{array} \end{array}\;\;\;\;\;\;\;\;\begin{array}{c} 0.45\\ 0.9\\ \begin{array}{c} 0.13\\ \begin{array}{c} 1.95\\ 0.1 \end{array} \end{array} \end{array}\;\;\;\;\;\;\;\;\begin{array}{c} 0.5\\ 0.85\\ \begin{array}{c} 0.19\\ \begin{array}{c} 2.85\\ 0.15 \end{array} \end{array} \end{array}\;\;\;\;\;\;\;\;\;\;\begin{array}{c} 0.55\\ 0.8\\ \begin{array}{c} 0.25\\ \begin{array}{c} 3.55\\ 0.2 \end{array} \end{array} \end{array}\;\;\;\;\;\;\;\;\;\;\begin{array}{c} 0.6\\ 0.75\\ \begin{array}{c} 0.3\\ \begin{array}{c} 4.1\\ 0.25 \end{array} \end{array} \end{array}\right)*\;\left(\begin{array}{c} 0.25\\ 0\\ \begin{array}{c} 0.25\\ \begin{array}{c} 0.25\\ 0.25 \end{array} \end{array} \end{array}\right)=\left(\begin{array}{c} 0.52125\\ 0.8375\\ \begin{array}{c} 0.21\\ \begin{array}{c} 3.0125\\ 0.1625 \end{array} \end{array} \end{array}\right)$$

For C_5_

$$\left(\begin{array}{c} Z_{1}\\ Z_{2}\\ \begin{array}{c} Z_{3}\\ \begin{array}{c} Z_{4}\\ Z_{5} \end{array} \end{array} \end{array}\right)=\left(\begin{array}{c} 0.435\\ 0.95\\ \begin{array}{c} 0.10\\ \begin{array}{c} 1.55\\ 0.05 \end{array} \end{array} \end{array}\;\;\;\;\;\;\;\;\begin{array}{c} 0.45\\ 0.9\\ \begin{array}{c} 0.13\\ \begin{array}{c} 1.95\\ 0.1 \end{array} \end{array} \end{array}\;\;\;\;\;\;\;\;\begin{array}{c} 0.5\\ 0.85\\ \begin{array}{c} 0.19\\ \begin{array}{c} 2.85\\ 0.15 \end{array} \end{array} \end{array}\;\;\;\;\;\;\;\;\;\;\begin{array}{c} 0.55\\ 0.8\\ \begin{array}{c} 0.25\\ \begin{array}{c} 3.55\\ 0.2 \end{array} \end{array} \end{array}\;\;\;\;\;\;\;\;\;\;\begin{array}{c} 0.6\\ 0.75\\ \begin{array}{c} 0.3\\ \begin{array}{c} 4.1\\ 0.25 \end{array} \end{array} \end{array}\right)*\;\left(\begin{array}{c} 0\\ 0.25\\ \begin{array}{c} 0.25\\ \begin{array}{c} 0.25\\ 0.25 \end{array} \end{array} \end{array}\right)=\left(\begin{array}{c} 0.525\\ 0.825\\ \begin{array}{c} 0.2175\\ \begin{array}{c} 3.1125\\ 0.175 \end{array} \end{array} \end{array}\right)$$

For C_12_

$$\left(\begin{array}{c} Z_{1}\\ Z_{2}\\ \begin{array}{c} Z_{3}\\ \begin{array}{c} Z_{4}\\ Z_{5} \end{array} \end{array} \end{array}\right)=\left(\begin{array}{c} 0.435\\ 0.95\\ \begin{array}{c} 0.10\\ \begin{array}{c} 1.55\\ 0.05 \end{array} \end{array} \end{array}\;\;\;\;\;\;\;\;\begin{array}{c} 0.45\\ 0.9\\ \begin{array}{c} 0.13\\ \begin{array}{c} 1.95\\ 0.1 \end{array} \end{array} \end{array}\;\;\;\;\;\;\;\;\begin{array}{c} 0.5\\ 0.85\\ \begin{array}{c} 0.19\\ \begin{array}{c} 2.85\\ 0.15 \end{array} \end{array} \end{array}\;\;\;\;\;\;\;\;\;\;\begin{array}{c} 0.55\\ 0.8\\ \begin{array}{c} 0.25\\ \begin{array}{c} 3.55\\ 0.2 \end{array} \end{array} \end{array}\;\;\;\;\;\;\;\;\;\;\begin{array}{c} 0.6\\ 0.75\\ \begin{array}{c} 0.3\\ \begin{array}{c} 4.1\\ 0.25 \end{array} \end{array} \end{array}\right)*\;\left(\begin{array}{c} 0.2\\ 0.2\\ \begin{array}{c} 0.2\\ \begin{array}{c} 0.2\\ 0.2 \end{array} \end{array} \end{array}\right)=\left(\begin{array}{c} 0.507\\ 0.85\\ \begin{array}{c} 0.194\\ \begin{array}{c} 2.8\\ 0.15 \end{array} \end{array} \end{array}\right)$$

For C_13_

$$\left(\begin{array}{c} Z_{1}\\ Z_{2}\\ \begin{array}{c} Z_{3}\\ \begin{array}{c} Z_{4}\\ Z_{5} \end{array} \end{array} \end{array}\right)=\left(\begin{array}{c} 0.435\\ 0.95\\ \begin{array}{c} 0.10\\ \begin{array}{c} 1.55\\ 0.05 \end{array} \end{array} \end{array}\;\;\;\;\;\;\;\;\begin{array}{c} 0.45\\ 0.9\\ \begin{array}{c} 0.13\\ \begin{array}{c} 1.95\\ 0.1 \end{array} \end{array} \end{array}\;\;\;\;\;\;\;\;\begin{array}{c} 0.5\\ 0.85\\ \begin{array}{c} 0.19\\ \begin{array}{c} 2.85\\ 0.15 \end{array} \end{array} \end{array}\;\;\;\;\;\;\;\;\;\;\begin{array}{c} 0.55\\ 0.8\\ \begin{array}{c} 0.25\\ \begin{array}{c} 3.55\\ 0.2 \end{array} \end{array} \end{array}\;\;\;\;\;\;\;\;\;\;\begin{array}{c} 0.6\\ 0.75\\ \begin{array}{c} 0.3\\ \begin{array}{c} 4.1\\ 0.25 \end{array} \end{array} \end{array}\right)*\;\left(\begin{array}{c} 0.3\\ 0.3\\ \begin{array}{c} 0.3\\ \begin{array}{c} 0.1\\ 0 \end{array} \end{array} \end{array}\right)=\left(\begin{array}{c} 0.4705\\ 0.89\\ \begin{array}{c} 0.151\\ \begin{array}{c} 2.26\\ 0.11 \end{array} \end{array} \end{array}\right)$$

For C_14_

$$\left(\begin{array}{c} Z_{1}\\ Z_{2}\\ \begin{array}{c} Z_{3}\\ \begin{array}{c} Z_{4}\\ Z_{5} \end{array} \end{array} \end{array}\right)=\left(\begin{array}{c} 0.435\\ 0.95\\ \begin{array}{c} 0.10\\ \begin{array}{c} 1.55\\ 0.05 \end{array} \end{array} \end{array}\;\;\;\;\;\;\;\;\begin{array}{c} 0.45\\ 0.9\\ \begin{array}{c} 0.13\\ \begin{array}{c} 1.95\\ 0.1 \end{array} \end{array} \end{array}\;\;\;\;\;\;\;\;\begin{array}{c} 0.5\\ 0.85\\ \begin{array}{c} 0.19\\ \begin{array}{c} 2.85\\ 0.15 \end{array} \end{array} \end{array}\;\;\;\;\;\;\;\;\;\;\begin{array}{c} 0.55\\ 0.8\\ \begin{array}{c} 0.25\\ \begin{array}{c} 3.55\\ 0.2 \end{array} \end{array} \end{array}\;\;\;\;\;\;\;\;\;\;\begin{array}{c} 0.6\\ 0.75\\ \begin{array}{c} 0.3\\ \begin{array}{c} 4.1\\ 0.25 \end{array} \end{array} \end{array}\right)*\;\left(\begin{array}{c} 0.3\\ 0.3\\ \begin{array}{c} 0.3\\ \begin{array}{c} 0\\ 0.1 \end{array} \end{array} \end{array}\right)=\left(\begin{array}{c} 0.4755\\ 0.885\\ \begin{array}{c} 0.156\\ \begin{array}{c} 2.315\\ 0.115 \end{array} \end{array} \end{array}\right)$$

For C_15_

$$\left(\begin{array}{c} Z_{1}\\ Z_{2}\\ \begin{array}{c} Z_{3}\\ \begin{array}{c} Z_{4}\\ Z_{5} \end{array} \end{array} \end{array}\right)=\left(\begin{array}{c} 0.435\\ 0.95\\ \begin{array}{c} 0.10\\ \begin{array}{c} 1.55\\ 0.05 \end{array} \end{array} \end{array}\;\;\;\;\;\;\;\;\begin{array}{c} 0.45\\ 0.9\\ \begin{array}{c} 0.13\\ \begin{array}{c} 1.95\\ 0.1 \end{array} \end{array} \end{array}\;\;\;\;\;\;\;\;\begin{array}{c} 0.5\\ 0.85\\ \begin{array}{c} 0.19\\ \begin{array}{c} 2.85\\ 0.15 \end{array} \end{array} \end{array}\;\;\;\;\;\;\;\;\;\;\begin{array}{c} 0.55\\ 0.8\\ \begin{array}{c} 0.25\\ \begin{array}{c} 3.55\\ 0.2 \end{array} \end{array} \end{array}\;\;\;\;\;\;\;\;\;\;\begin{array}{c} 0.6\\ 0.75\\ \begin{array}{c} 0.3\\ \begin{array}{c} 4.1\\ 0.25 \end{array} \end{array} \end{array}\right)*\;\left(\begin{array}{c} 0.3\\ 0.3\\ \begin{array}{c} 0\\ \begin{array}{c} 0.3\\ 0.1 \end{array} \end{array} \end{array}\right)=\left(\begin{array}{c} 0.4905\\ 0.87\\ \begin{array}{c} 0.174\\ \begin{array}{c} 2.525\\ 0.13 \end{array} \end{array} \end{array}\right)$$

For C_23_

$$\left(\begin{array}{c} Z_{1}\\ Z_{2}\\ \begin{array}{c} Z_{3}\\ \begin{array}{c} Z_{4}\\ Z_{5} \end{array} \end{array} \end{array}\right)=\left(\begin{array}{c} 0.435\\ 0.95\\ \begin{array}{c} 0.10\\ \begin{array}{c} 1.55\\ 0.05 \end{array} \end{array} \end{array}\;\;\;\;\;\;\;\;\begin{array}{c} 0.45\\ 0.9\\ \begin{array}{c} 0.13\\ \begin{array}{c} 1.95\\ 0.1 \end{array} \end{array} \end{array}\;\;\;\;\;\;\;\;\begin{array}{c} 0.5\\ 0.85\\ \begin{array}{c} 0.19\\ \begin{array}{c} 2.85\\ 0.15 \end{array} \end{array} \end{array}\;\;\;\;\;\;\;\;\;\;\begin{array}{c} 0.55\\ 0.8\\ \begin{array}{c} 0.25\\ \begin{array}{c} 3.55\\ 0.2 \end{array} \end{array} \end{array}\;\;\;\;\;\;\;\;\;\;\begin{array}{c} 0.6\\ 0.75\\ \begin{array}{c} 0.3\\ \begin{array}{c} 4.1\\ 0.25 \end{array} \end{array} \end{array}\right)*\;\left(\begin{array}{c} 0.3\\ 0\\ \begin{array}{c} 0.3\\ \begin{array}{c} 0.3\\ 0.1 \end{array} \end{array} \end{array}\right)=\left(\begin{array}{c} 0.5055\\ 0.855\\ \begin{array}{c} 0.192\\ \begin{array}{c} 2.795\\ 0.145 \end{array} \end{array} \end{array}\right)$$

For C_24_

$$\left(\begin{array}{c} Z_{1}\\ Z_{2}\\ \begin{array}{c} Z_{3}\\ \begin{array}{c} Z_{4}\\ Z_{5} \end{array} \end{array} \end{array}\right)=\left(\begin{array}{c} 0.435\\ 0.95\\ \begin{array}{c} 0.10\\ \begin{array}{c} 1.55\\ 0.05 \end{array} \end{array} \end{array}\;\;\;\;\;\;\;\;\begin{array}{c} 0.45\\ 0.9\\ \begin{array}{c} 0.13\\ \begin{array}{c} 1.95\\ 0.1 \end{array} \end{array} \end{array}\;\;\;\;\;\;\;\;\begin{array}{c} 0.5\\ 0.85\\ \begin{array}{c} 0.19\\ \begin{array}{c} 2.85\\ 0.15 \end{array} \end{array} \end{array}\;\;\;\;\;\;\;\;\;\;\begin{array}{c} 0.55\\ 0.8\\ \begin{array}{c} 0.25\\ \begin{array}{c} 3.55\\ 0.2 \end{array} \end{array} \end{array}\;\;\;\;\;\;\;\;\;\;\begin{array}{c} 0.6\\ 0.75\\ \begin{array}{c} 0.3\\ \begin{array}{c} 4.1\\ 0.25 \end{array} \end{array} \end{array}\right)*\;\left(\begin{array}{c} 0\\ 0.3\\ \begin{array}{c} 0.3\\ \begin{array}{c} 0.3\\ 0.1 \end{array} \end{array} \end{array}\right)=\left(\begin{array}{c} 0.51\\ 0.84\\ \begin{array}{c} 0.201\\ \begin{array}{c} 2.915\\ 0.16 \end{array} \end{array} \end{array}\right)$$

For C_25_

$$\left(\begin{array}{c} Z_{1}\\ Z_{2}\\ \begin{array}{c} Z_{3}\\ \begin{array}{c} Z_{4}\\ Z_{5} \end{array} \end{array} \end{array}\right)=\left(\begin{array}{c} 0.435\\ 0.95\\ \begin{array}{c} 0.10\\ \begin{array}{c} 1.55\\ 0.05 \end{array} \end{array} \end{array}\;\;\;\;\;\;\;\;\begin{array}{c} 0.45\\ 0.9\\ \begin{array}{c} 0.13\\ \begin{array}{c} 1.95\\ 0.1 \end{array} \end{array} \end{array}\;\;\;\;\;\;\;\;\begin{array}{c} 0.5\\ 0.85\\ \begin{array}{c} 0.19\\ \begin{array}{c} 2.85\\ 0.15 \end{array} \end{array} \end{array}\;\;\;\;\;\;\;\;\;\;\begin{array}{c} 0.55\\ 0.8\\ \begin{array}{c} 0.25\\ \begin{array}{c} 3.55\\ 0.2 \end{array} \end{array} \end{array}\;\;\;\;\;\;\;\;\;\;\begin{array}{c} 0.6\\ 0.75\\ \begin{array}{c} 0.3\\ \begin{array}{c} 4.1\\ 0.25 \end{array} \end{array} \end{array}\right)*\;\left(\begin{array}{c} 0.1\\ 0\\ \begin{array}{c} 0.3\\ \begin{array}{c} 0.3\\ 0.3 \end{array} \end{array} \end{array}\right)=\left(\begin{array}{c} 0.5385\\ 0.815\\ \begin{array}{c} 0.232\\ \begin{array}{c} 3.305\\ 0.185 \end{array} \end{array} \end{array}\right)$$

For C_34_

$$\left(\begin{array}{c} Z_{1}\\ Z_{2}\\ \begin{array}{c} Z_{3}\\ \begin{array}{c} Z_{4}\\ Z_{5} \end{array} \end{array} \end{array}\right)=\left(\begin{array}{c} 0.435\\ 0.95\\ \begin{array}{c} 0.10\\ \begin{array}{c} 1.55\\ 0.05 \end{array} \end{array} \end{array}\;\;\;\;\;\;\;\;\begin{array}{c} 0.45\\ 0.9\\ \begin{array}{c} 0.13\\ \begin{array}{c} 1.95\\ 0.1 \end{array} \end{array} \end{array}\;\;\;\;\;\;\;\;\begin{array}{c} 0.5\\ 0.85\\ \begin{array}{c} 0.19\\ \begin{array}{c} 2.85\\ 0.15 \end{array} \end{array} \end{array}\;\;\;\;\;\;\;\;\;\;\begin{array}{c} 0.55\\ 0.8\\ \begin{array}{c} 0.25\\ \begin{array}{c} 3.55\\ 0.2 \end{array} \end{array} \end{array}\;\;\;\;\;\;\;\;\;\;\begin{array}{c} 0.6\\ 0.75\\ \begin{array}{c} 0.3\\ \begin{array}{c} 4.1\\ 0.25 \end{array} \end{array} \end{array}\right)*\;\left(\begin{array}{c} 0.1\\ 0.3\\ \begin{array}{c} 0\\ \begin{array}{c} 0.3\\ 0.3 \end{array} \end{array} \end{array}\right)=\left(\begin{array}{c} 0.5235\\ 0.83\\ \begin{array}{c} 0.214\\ \begin{array}{c} 3.035\\ 0.155 \end{array} \end{array} \end{array}\right)$$

For C_35_

$$\left(\begin{array}{c} Z_{1}\\ Z_{2}\\ \begin{array}{c} Z_{3}\\ \begin{array}{c} Z_{4}\\ Z_{5} \end{array} \end{array} \end{array}\right)=\left(\begin{array}{c} 0.435\\ 0.95\\ \begin{array}{c} 0.10\\ \begin{array}{c} 1.55\\ 0.05 \end{array} \end{array} \end{array}\;\;\;\;\;\;\;\;\begin{array}{c} 0.45\\ 0.9\\ \begin{array}{c} 0.13\\ \begin{array}{c} 1.95\\ 0.1 \end{array} \end{array} \end{array}\;\;\;\;\;\;\;\;\begin{array}{c} 0.5\\ 0.85\\ \begin{array}{c} 0.19\\ \begin{array}{c} 2.85\\ 0.15 \end{array} \end{array} \end{array}\;\;\;\;\;\;\;\;\;\;\begin{array}{c} 0.55\\ 0.8\\ \begin{array}{c} 0.25\\ \begin{array}{c} 3.55\\ 0.2 \end{array} \end{array} \end{array}\;\;\;\;\;\;\;\;\;\;\begin{array}{c} 0.6\\ 0.75\\ \begin{array}{c} 0.3\\ \begin{array}{c} 4.1\\ 0.25 \end{array} \end{array} \end{array}\right)*\;\left(\begin{array}{c} 0.1\\ 0.3\\ \begin{array}{c} 0.3\\ \begin{array}{c} 0\\ 0.3 \end{array} \end{array} \end{array}\right)=\left(\begin{array}{c} 0.5085\\ 0.845\\ \begin{array}{c} 0.196\\ \begin{array}{c} 2.825\\ 0.155 \end{array} \end{array} \end{array}\right)$$

For C_45_

$$\left(\begin{array}{c} Z_{1}\\ Z_{2}\\ \begin{array}{c} Z_{3}\\ \begin{array}{c} Z_{4}\\ Z_{5} \end{array} \end{array} \end{array}\right)=\left(\begin{array}{c} 0.435\\ 0.95\\ \begin{array}{c} 0.10\\ \begin{array}{c} 1.55\\ 0.05 \end{array} \end{array} \end{array}\;\;\;\;\;\;\;\;\begin{array}{c} 0.45\\ 0.9\\ \begin{array}{c} 0.13\\ \begin{array}{c} 1.95\\ 0.1 \end{array} \end{array} \end{array}\;\;\;\;\;\;\;\;\begin{array}{c} 0.5\\ 0.85\\ \begin{array}{c} 0.19\\ \begin{array}{c} 2.85\\ 0.15 \end{array} \end{array} \end{array}\;\;\;\;\;\;\;\;\;\;\begin{array}{c} 0.55\\ 0.8\\ \begin{array}{c} 0.25\\ \begin{array}{c} 3.55\\ 0.2 \end{array} \end{array} \end{array}\;\;\;\;\;\;\;\;\;\;\begin{array}{c} 0.6\\ 0.75\\ \begin{array}{c} 0.3\\ \begin{array}{c} 4.1\\ 0.25 \end{array} \end{array} \end{array}\right)*\;\left(\begin{array}{c} 0.1\\ 0.3\\ \begin{array}{c} 0.3\\ \begin{array}{c} 0.3\\ 0 \end{array} \end{array} \end{array}\right)=\left(\begin{array}{c} 0.4935\\ 0.86\\ \begin{array}{c} 0.181\\ \begin{array}{c} 2.66\\ 0.14 \end{array} \end{array} \end{array}\right)$$

The matrix table for the mixture proportion formulation is presented in Table 2.

### 2.5. Chemical Characterization

This is a vital procedure in material science that describes the broad and general method of probing and measuring a material’s structure and properties [18].

- X-ray fluorescence (XRF) is a secondary characteristic emission from a substance fraught by being barraged with high-energy gamma or X-rays. The trend is broadly adapted for the assessment of chemical and elemental oxides, for the proper characterization of the test materials’ chemical constituents and for research in geochemistry and forensic science [51]. High-energy photons are used in XRF spectroscopy to bombard an atom so as to excite the electrons around it. Several photons are created with enough energy to expel an electron attached to the atom’s nucleus. When an electron from an atom’s inner orbital is evicted, an electron from a higher energy orbital is moved to the lower energy orbital, causing the atom to produce X-rays or photons in a process known as fluorescence [52].
- Scanning electron microscopy (SEM) uses a kind of electron microscope to directly study the surfaces of solid objects or materials through the utilization of a beam of the directed electrons of relatively low energy scanned in a regular manner over the surface of the specimen. Large, hardened and bulky specimen can be taken for investigation in the SEM as no specific sample preparation technique is required. For clear imaging to be obtained, the specimen to be tested needs to be electrically conducting. To achieve this level of conductivity, a film of a metal such as gold of 50–100 Angstroms thick is evaporated on the surface of the specimen in a vacuum [53].
- In EDXRF spectrometers, a sample is directly irradiated by an X-ray tube functioning as a source, and the fluorescence emitted by the sample is detected with an energy dispersive detector. All spectrometers have three basic components: a radiation source, sample substance and detection mechanism. The varied energy of the characteristic radiation coming straight from the sample can be measured using this detector. The detector can distinguish between the radiation emitted by the sample and the radiation emitted by the various elements present in the sample. Dispersion is the term for this separation [50]. The sample is produced, mounted on a stud and inserted into the chamber of a machine with an SEM capability to obtain such an image. As needed, the technician can move the observation lens around and focus on different places. Under various magnifications, a variety of images can be created. The elements that predominate in the sample can also be determined using energy-dispersive X-ray spectroscopy (EDS). Comparing the attributes of known specimens can help determine the sample’s elemental and microstructural composition [54].

### 2.6. Permeability Tests

Permeability analysis on pervious concrete specimens was also performed using the falling head method, which involves sealing the specimen and placing it between two pipes. The time it takes for the water pressure head to drop to preset values (*h*_1_ and *h*_2_) was recorded and utilized in Equation (16) to compute the hydraulic conductivity (*k*) of the pervious concrete mixtures using the mathematical expression provided in Equation (16). Where *A* and *L* are the cross-sectional area and length of the specimen, respectively, *a* is the cross-sectional area of the pipe shrouding the specimen [55,56].

(17) $$k=\frac{aL}{At}\ln\frac{h_{1}}{h_{2}}$$

### 2.7. Cantabro Test (ASTM C1747)

The Cantabro test is a fast and intensive method of evaluating pervious concrete durability that involves impact loading a half-height 100 mm diameter cylinder in a rotational steel drum. The mass loss should be kept to a minimum. The test can be halted at predetermined intervals (after 50 or 100 cycles) to capture the intermediate mass loss and observe the damage progression as well as the ultimate mass loss value after 500 revolutions. ASTM C944 is an accelerated test technique for determining the abrasion resistance of pervious concrete. Modifying a press drill to hold a rotary cutter made of stacked washers of 25–32 in diameter revolving at a speed of 200 rounds per minute under a load of roughly 98 N or a doubling load creates the test equipment. The test is carried out on three different regions that replicate the pervious concrete pavement’s surface [57].

### 2.8. Flexural Strength Test

**Figure 3:**
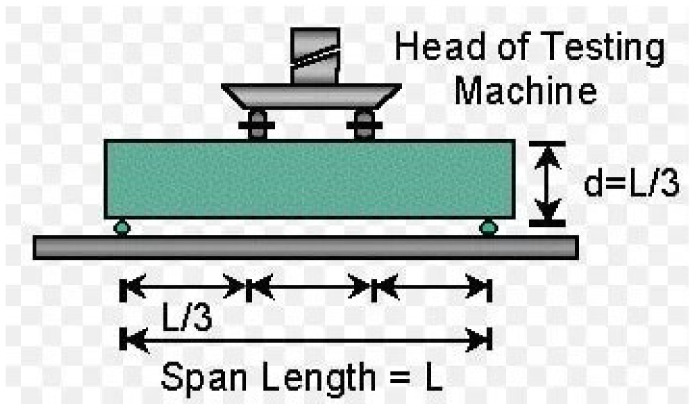
Loading Configuration: Four-Point Load Flexural Test (ASTM C78).

This test laboratory method helps assess the rupture or bending strength of the concrete material derived just before the test sample under study yields in a flexure test. The derived test response is the maximum stress that the concrete material experiences within its yield moment. The concrete samples for this test are thoroughly mixed and compacted in a beam molds with dimensions of 100 mm × 100 mm × 400 mm. The resulting concrete beams were demolded and cured for a 28 day period of hydration before being taken for the flexural test. Three replicates for each Scheffe’s experimental runs were produced with forty-five concrete beams for an experimental test utilized for the model formulation, while the other forty-five beams (control test) were taken to evaluate the developed Scheffe’s regression model. After 28 days of curing, the three samplings for each mixture were crushed and the average flexural strength was calculated using the calculation in Equation (17). The flexural testing configuration is presented in Figure 3 [58,59].

(18) $$\sigma =\frac{FL}{bd^{2}}$$

### 2.9. Model Statistical Test of Adequacy

The adequacy test of the generated Scheffe model was carried out using a statistical method, namely Student’s *t*-test, as well as an analysis of variance (ANOVA), which is utilized to determine the mean differences between the control experiment or actual results and the model-predicted results. With respect to the flexural strength responses, statistical tests are conducted for second-order regression models developed at a 95% confidence level. By substituting the respective values of the pseudo-components (X_i_) into the created model equation, the predicted values (Y-predicted) for the test control points were obtained [34,60].

- Null Hypothesis

There is no considerable variance between the model-predicted and laboratory test results.

- Alternative Hypothesis

The findings of the laboratory test and those anticipated by the model are significantly different.

## 3. Results Discussion and Analysis

### 3.1. Physicochemical Properties of the Test Materials

**Figure 4:**
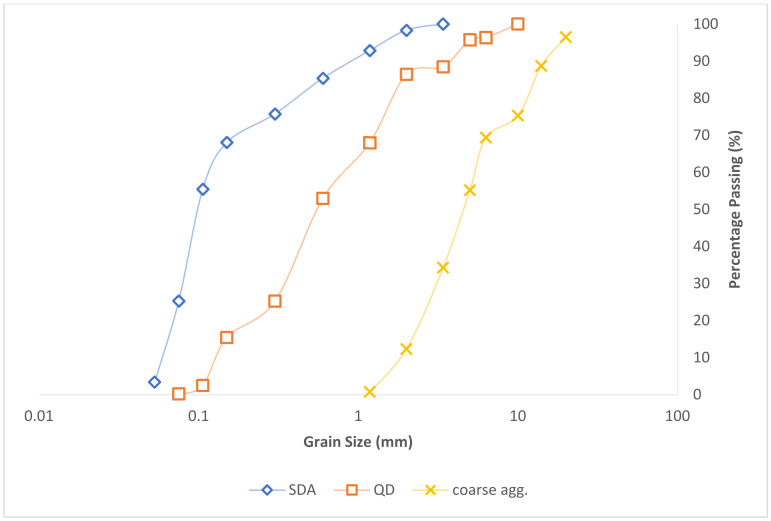
Particle Size Distribution Plot.

Series of laboratory tests were carried out on the mixture components to evaluate their general engineering behavior as civil construction materials. Sieve analysis and specific gravity tests were carried out on the test aggregates and admixtures to assess the gradation and particle size distribution. The sieve analysis test result which illustrates the variation in the soil grain sizes using a cumulative frequency distribution curve is shown in the semi-log graph in Figure 4. From the obtained results, 75.3–12.3% are the passing sieve size of 10–2 mm for the coarse aggregate. For the admixtures QD and SDA, 86.48–0.23% and 98.37–25.32% are the passing through sieve size of 2 mm–75 µm, respectively. The coefficients of gradation computation are further presented in Table 3, and the obtained results indicate well-graded sand and gravel particles that also fall within the requirements specified by BS 882 for improved concrete durability performance [61,62].

### 3.2. Chemical Characterization of the Test Cement, SDA and QD

The assessment of the chemical properties of the test admixtures was achieved using X-ray fluorescence (XRF). The obtained result showed that SDA mostly has SiO_2_ (57.85%), Al_2_O_3_ (8.35%) and Fe_2_O_3_ (4.3%), which produces a sum of 70.52% by composition, whilst QD on the other hand possesses SiO_2_ (48.5%), Al_2_O_3_ (15.93%) and Fe_2_O_3_ (6.01%) to also produce a total sum of 70.44% by composition which showed a good pozzolanic property in accordance with ASTM C618, 98 specifications. The abundance of calcium oxide in the test materials—13.52%, 10.4% and 11.3% derived from QD, SDA and Portland cement binder, respectively—enhances the complete cement hydration which improves the mechanical strength and durability behavior of the green concrete produced, as presented in Table 4 and Table 5. The hydration reaction mechanism enables the oxides of aluminate and silicates obtained from the admixture blends with hydrated calcium (lime) to generate hydration products which form a harder mass with time. The results of the physical properties indicate the bulk density and specific gravity of 946 kg/m^3^, 2.24 and 1755 kg/m^3^, 2.62 for SDA and QD, respectively [63,64].

### 3.3. Slump Test Results

**Figure 5:**
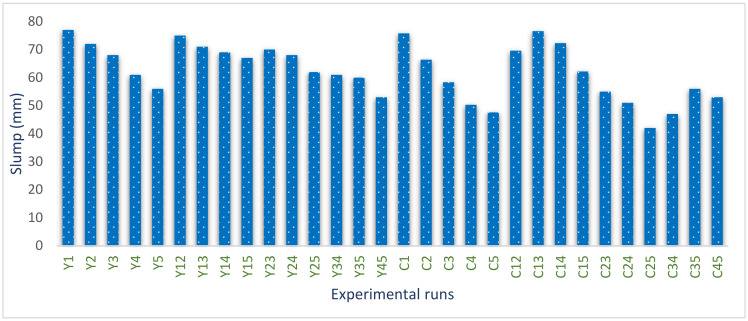
Slump test plotted result.

**Figure 6:**
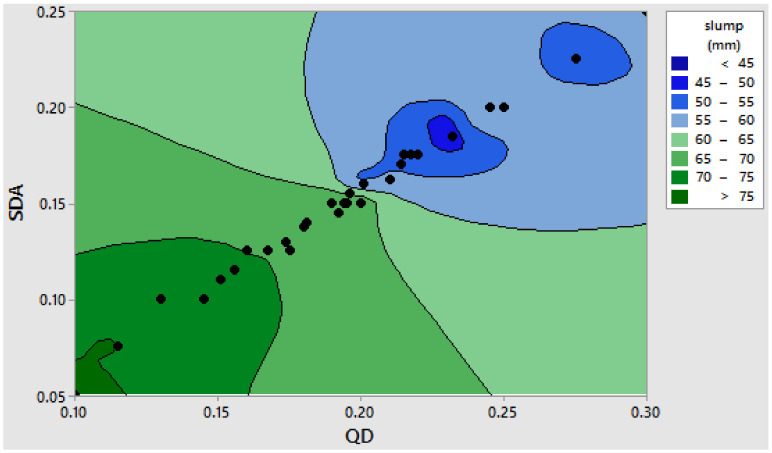
Contour plot of SDA and QD vs. slump response. (The black dots illustrate the regions in the legend where the points for the SDA and the QD factors meet. The response magnitude at those points are shown in the legend in varying color forms).

Laboratory tests to assess the workability properties of the freshly mixed blended cement–SDA–QD concrete matrix were carried out. This test aimed to determine the placeability and workability behavior of the fresh concrete mixture with respect to the erratic ratios of cement–SDA and fine agg.–QD combinations as defined by Scheffe’s mixture design formulation. The obtained experimental results indicate that the value of the slump test reduces with the increase in the QD and SDA fractions in the concrete mixture, thereby resulting in more water being needed in order to make the mixture more workable. The reason may be attributed to the presence of alumino-silica content in the admixtures as well as the surface area increment (Mohammed et al., 2012). The obtained result is presented in a contour plot assessing the impact of the admixtures on the workability behavior of the concrete, as shown in Figure 5 and Figure 6. The derived results indicate that the Y_1_ experimental point with 14.1% water, 30.8% cement, 3.25% QD, 50.243% coarse aggregate and 1.62% SDA produced a maximum slump value of 77 mm. However, the C_12_ experimental point produced a minimum slump value of 42 mm with 10.61% water, 16.06% cement, 4.6% QD, 50.243% coarse aggregate and 3.645% SDA. The obtained results showed a linear decrement in the slump response of the freshly blended concrete specimen as the percentages of the admixtures present in the mix increases [65,66]

### 3.4. Abrasion Resistance Test

**Figure 7:**
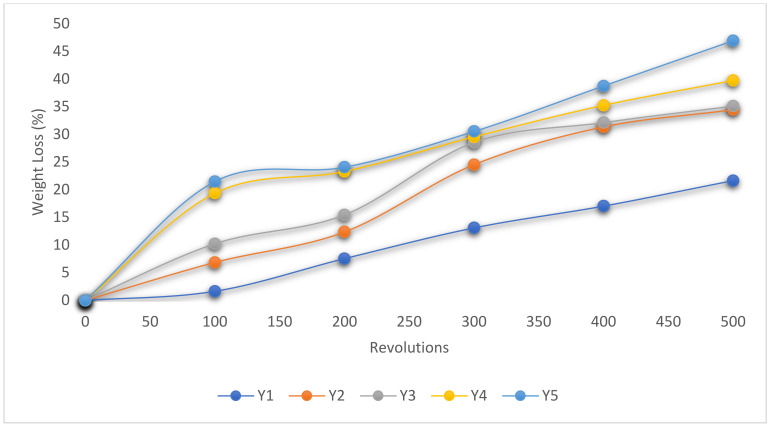
Abrasion resistance test result.

To examine the abrasion resistance behavior of the pervious concrete produced, the hardened cylindrical concrete samples for the five pure blends achieved in the design experimental points were taken for the experiment. The test concrete samples were first weighed to derive its initial weight and taken to the Cantabro test apparatus to assess the rate of mass loss after each of the 100 revolutions, with the overall results taken and plotted after a total of 500 revolutions, as presented in Figure 7. The obtained laboratory response calculated showed that Y_1_ produced a minimum weight loss of 1.64–21.64% from 100–500 revolutions, respectively [67]. The weight loss was observed to linearly increase as the content fractions of SDA and QD increase with the maximum result obtained at Y_5_ with a weight loss of 21.48–46.94% from 100 to 500 revolutions, respectively. Wu et al. [57] found nearly identical results. After 300 revolutions, they discovered that 20% of the weight of the pervious concrete samples had been lost.

### 3.5. Hydraulic Conductivity

**Figure 8:**
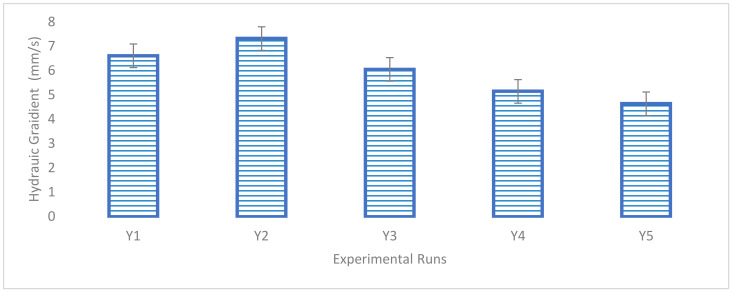
Hydraulic conductivity results.

The hydraulic conductivity property is essential as it is required to enable the surface runoff to infuse through the pervious concrete surface and be directed to the safe discharge point to avert flooding and erosion issues. The hydraulic conductivity property testing provides an essential evaluation parameter to define the penetrability of pervious concrete, which would enable the proper channeling of surface runoff from the road way to avoid failure due to erosion. Water must be able to travel through a pervious concrete pavement at a rate of at least 5.4 mm/s [68,69]. From the experimental results obtained using the falling head apparatus for the first five binary blend points in Scheffe’s factor space, a maximum permeability for Y_2_ at 7.32 mm/s with was obtained with SDA and QD contents of 2.833% and 3.683%, respectively, while a minimum response of 4.64 mm/s was obtained at Y_5_ with SDA and QD contents of 4.167% and 5.0%, respectively, as shown in the graphical bar chart in Figure 8. The resulting experimental results reveal that, as the ratio of SDA and QD grows, the hydraulic conductivity value linearly decreases, indicating that the pervious concrete voids are higher at minimum SDA and QD contents in the mixture of 1.5–3.0% and 3.2–3.7%, respectively. These findings matched those of Tennis et al. [13], who found that the usual range of permeability values for pervious concrete was between 2 mm/s and 12 mm/s.

### 3.6. Flexural Strength Result

**Figure 9:**
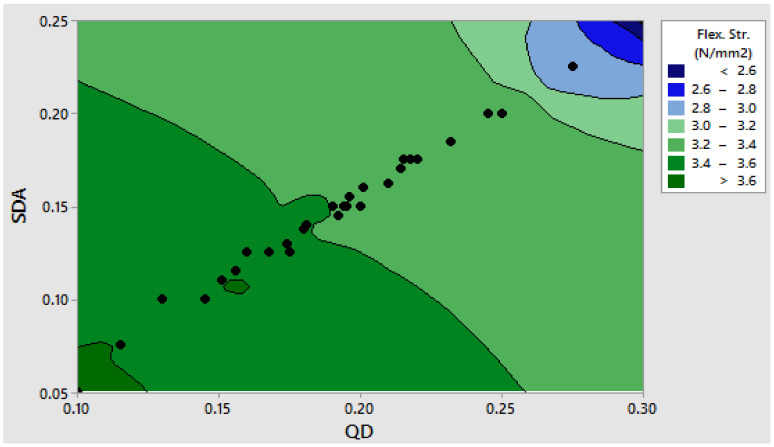
Contour plot for the flexural strength response vs. admixtures. (The black dots illustrate the regions in the legend where the points for the SDA and the QD factors meet. The response magnitude at those points are shown in the legend in varying color forms).

**Figure 10:**
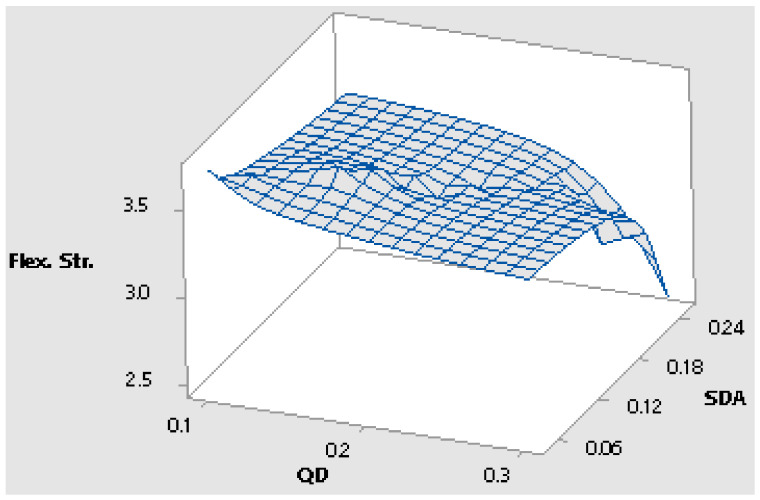
Three-dimensional surface plot of flexural strength response vs. admixtures.

For the fifteen distinct combination design points, experimental results in terms of the flexural strength of concrete beam samples hydrated for twenty-eight days were obtained with a total of three replicates for each design point. The values are used to generate Scheffe’s second-order regression model for the compressive strength property of pervious concrete optimization using SDA and QD additives [70]. As indicated in Table 6, the experimental response corresponding to Y_1_ produced the greatest strength value of 3.703 MPa, while points corresponding to Y_5_ produced the minimum values of 2.504 MPa. Table 7 also includes the laboratory answers for the control points used to validate Scheffe’s second-order regression model. The maximum flexural strength value was 3.681 MPa for the points corresponding to C_1_, while the minimum flexural strength value was 3.193 MPa for sites corresponding to C45 [22,58]. Due to the addition of SDA and QD admixtures to the concrete matrix, these results imply an improved performance in terms of the concrete’s strength property. Figure 9 and Figure 10 show a 3D surface and contour plot depicting the influence of admixtures on the pervious concrete’s flexural strength response [71].

#### Scheffe’s Regression Equation

By the substitution of the obtained laboratory responses for the concrete’s flexural strength property in Equation (14) into Equation (12), the model equation is as shown in Equation (18) and Table 8. The computer program for the model development performed using MATLAB R2020a computational software and the command script is presented in the Supplementary Materials.

(19) $$\hat{Y}=3.70X_{1}+3.53X_{2}+3.61X_{3}+3.29X_{4}+2.50X_{5}-0.09X_{1}X_{2}-0.61X_{1}X_{3}-0.19X_{1}X_{4}+\;0.08X_{1}X_{5}-0.57X_{2}X_{3}-0.35X_{2}X_{4}+0.87X_{2}X_{5}-0.82X_{3}X_{4}+0.56X_{3}X_{5}-0.30X_{4}X_{5}$$

### 3.7. Test of Adequacy and Validation of Scheffe’s Model

**Figure 11:**
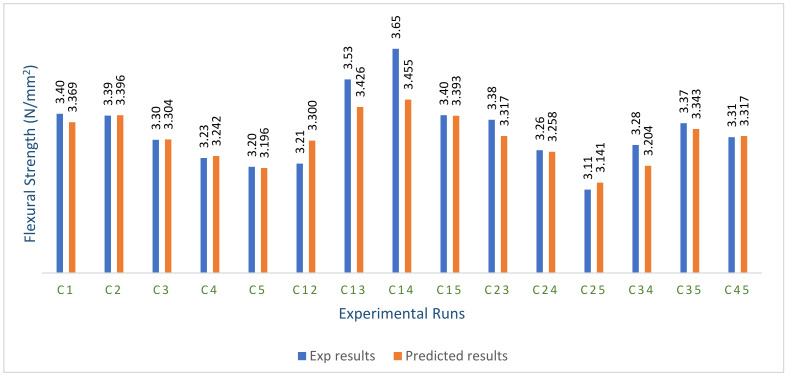
Scheffe’s model results vs. experimental control points for flexural strength.

Using Student’s *t*-test and ANOVA, the adequacy of the constructed Scheffe’s model was tested using the experiment’s control points. Figure 11 illustrates the experimental or actual control laboratory flexural responses, as well as the values obtained from the developed Scheffe’s simplex-lattice quadratic model [72].

#### Evaluation of Scheffe’s Model for Flexural Strength Property

The developed second-order regression model’s prediction performance was evaluated using an analysis of variance (ANOVA) by statistically assessing the laboratory- and model-predicted values at 95% confidence intervals, as shown in Table 9 using the stated condition; if F > F crit, the null hypothesis is rejected. From the statistical computation, F = 0.359 and F crit = 4.195, indicating that F crit > F; therefore, we accept that the null hypothesis has a *p*-value of 0.554, which is greater than the alpha value of 0.05. This indicates that there was no significant difference between the actual or laboratory-derived results and the model-predicted results [73,74].

We used the aforementioned requirement to conduct a two-tail inequality *t*-test to further assess the created model prediction performance; if t Stat > t critical two-tail, we reject the null hypothesis. Table 10 shows a t stat of 1.502 and a t critical two-tail of 2.145, indicating that t critical > t stat. As a result, we accept the null hypothesis because the two-tail P(T = t) of 0.1554 is greater than the critical value of 0.05. As a result, the created model can be used to forecast the flexural properties of green pervious concrete [75,76].

### 3.8. Sensitivity Analysis

The sensitivity analysis was performed to evaluate the impact of each factor level or model input parameter on the target variable. In this case, the input variables are the components of a five-component mixture design optimization based on Scheffe’s theory. A unitary input variable is deleted for a specific trial test to perform a sensitivity analysis for the constructed quadratic regression model, and a regression model is built to generalize the remaining components with the output variable. The mean absolute error (MAE) and root mean squared error (RMSE) loss function parameters were used to statistically assess the model performance [77,78]. This procedure is continued until all of the input variables were eliminated and a regression model was built for all of the instances. As indicated in Table 11, the trial with the highest calculated loss function score was chosen as the most sensitive parameter. The derived statistical results indicate that the water–cement ratio (w/c) was the most influential factor with a maximum RMSE and MAE of 4.51 and 4.17, respectively [79].

### 3.9. Microstructural Characterization

**Figure 12:**
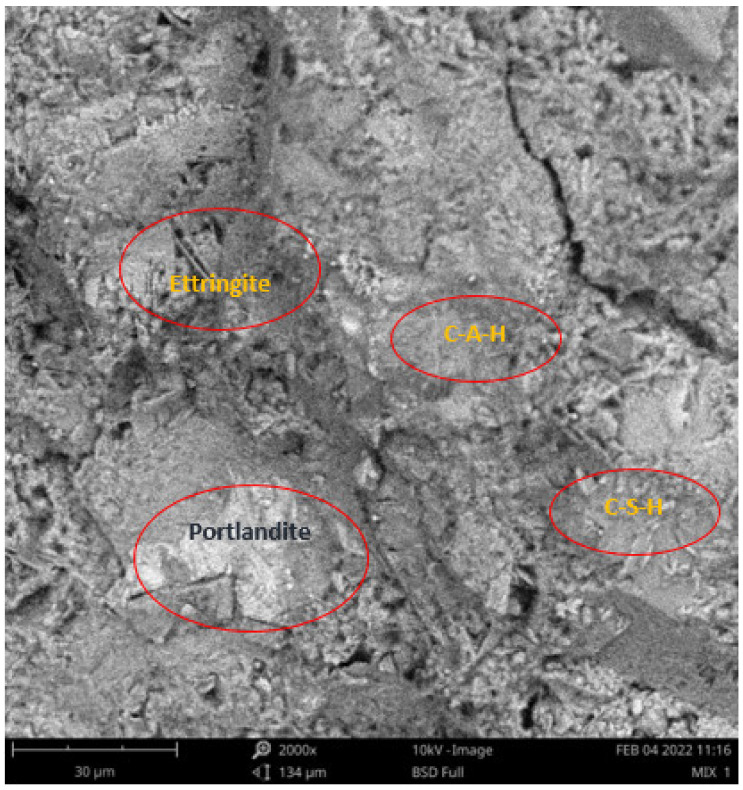
Micrograph of Y_1_ concrete specimen.

**Figure 13:**
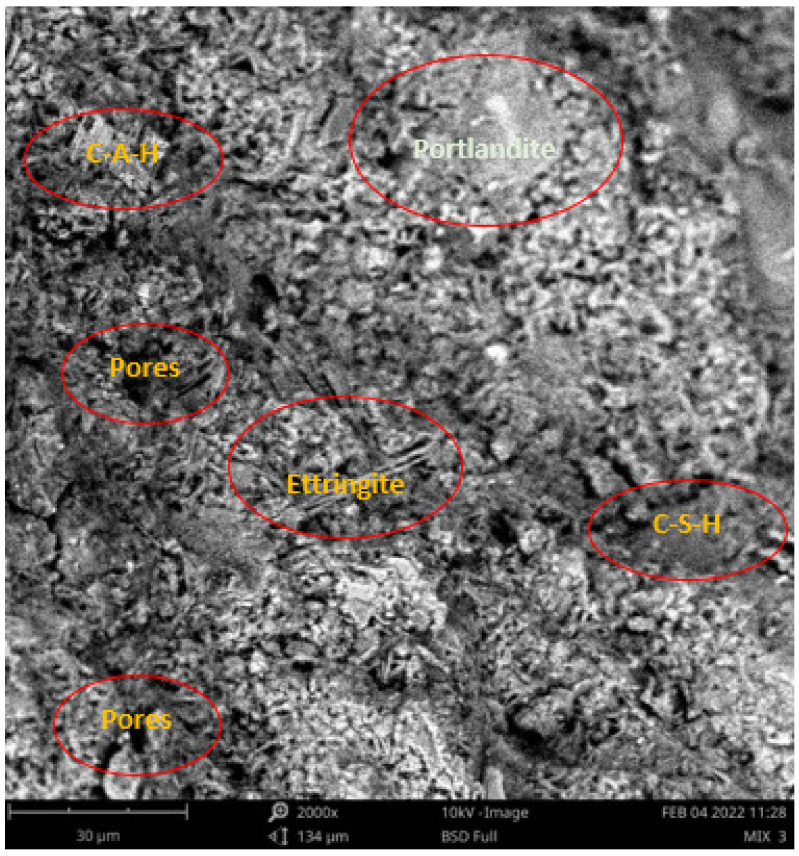
Micrograph of Y_3_ concrete specimen.

**Figure 14:**
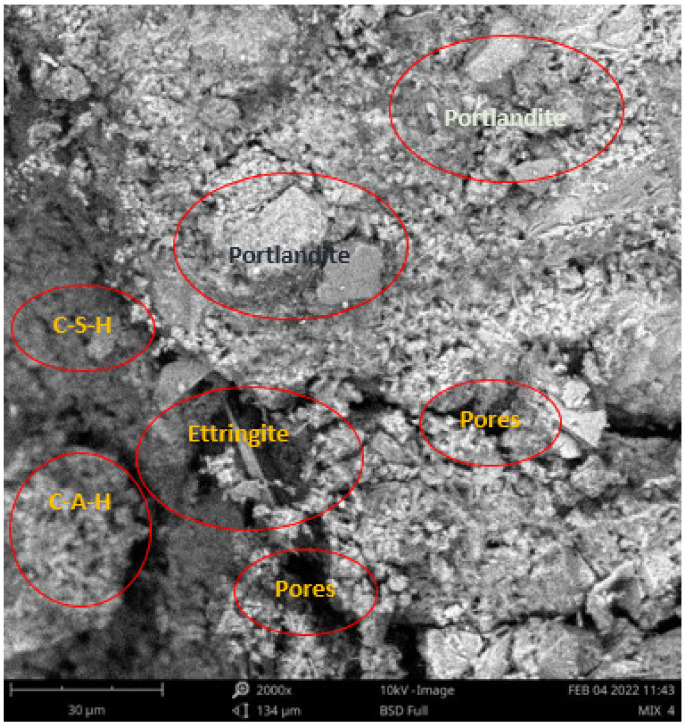
Micrograph of Y_4_ concrete specimen.

Scanning electron microscope (SEM) and energy dispersive spectroscopy (EDS) were deployed for the assessment of the microstructural and morphological performance of the QD and SDA blended with pervious concrete specimens. Taking a critical examination of the experimental points for the binary blends with a maximum and minimum rheological and mechanical strength performance (Y_1_, Y_3_ and Y_4_) served to designate mix 1, mix 3 and mix 4, respectively. These nano-tests were performed in order to elucidate and ratify the pozzolanic action from the morphological examination of the concrete specimens as a result of the optimization exercise shown in Figure 12, Figure 13 and Figure 14 [18,48].

Micrographs revealed that mix 1 had a smoother and more compacted surface, whereas mixes 3 and 4 had rough-surface morphologies, which could be due to a change in the orientation and fabric of the blended concrete matrix as a result of a bigger SDA and QD admixture content, resulting in high porosity due to the texture, size, and shape of QD particles. These loose voids observed in the micrograph illustrate the effects of a lack of fine aggregates in the concrete specimen which allows the easy permeation of water through the surface. However, due to the pozzolanic reaction, QD is added to combine with the coarse aggregates, whereas SDA combines with cement in the matrix to generate the precipitate of new cementitious compounds such as calcium aluminate hydrate CAH and calcium silicate hydrate CSH. These findings are consistent with previous research [62,80], which found that their microstructural alterations contribute to strength growth over time.

#### SEM-EDS Analysis

**Figure 15:**
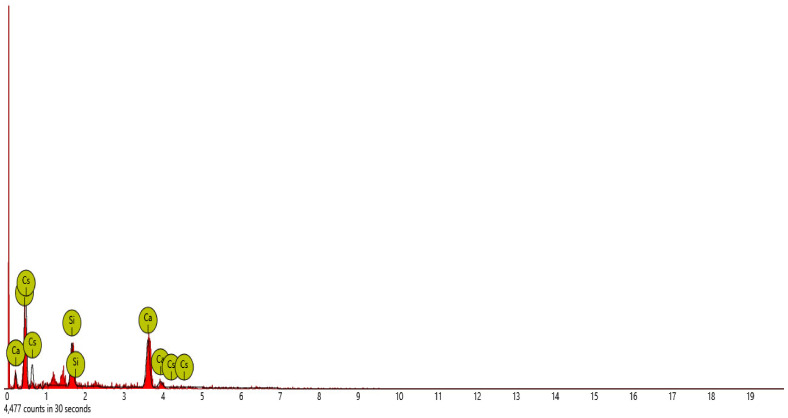
Graphical charts illustrating the atomic quantifications and weights of the elements discovered from the EDS spectra of pervious concrete spot 1 sample Y_1_.

**Figure 16:**
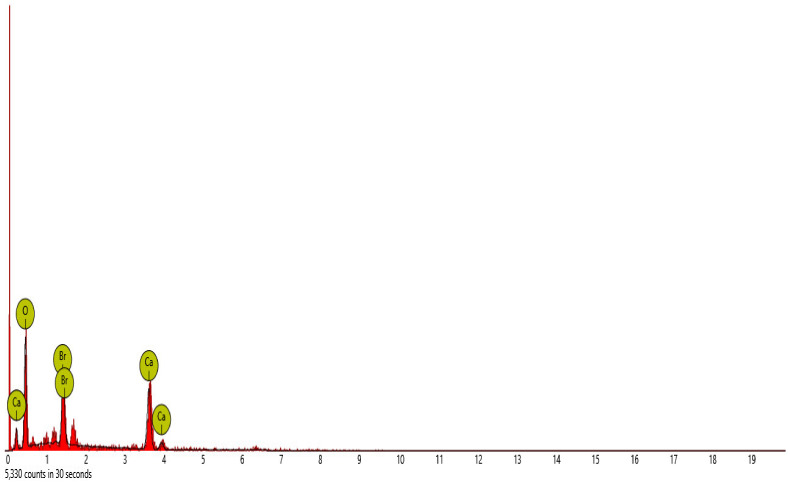
Graphical charts illustrating the atomic quantifications and weights of the elements discovered from the EDS spectra of pervious concrete spot 2 sample Y_1_.

**Figure 17:**
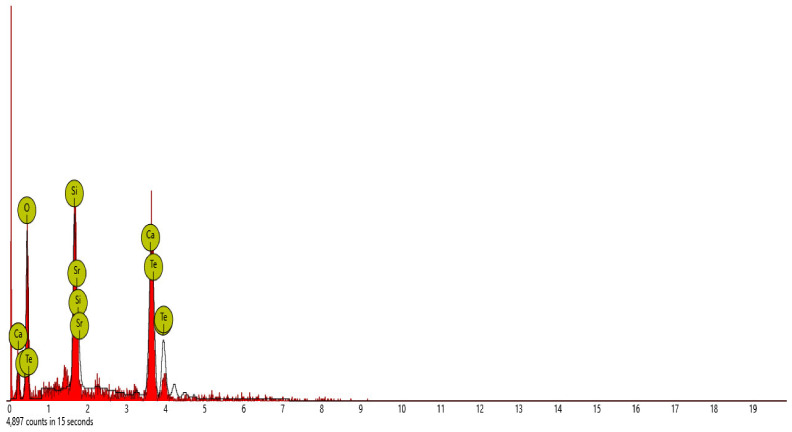
Graphical charts illustrating the atomic quantifications and the weight of the elements discovered from the EDS spectra of pervious concrete sample Y_3_.

**Figure 18:**
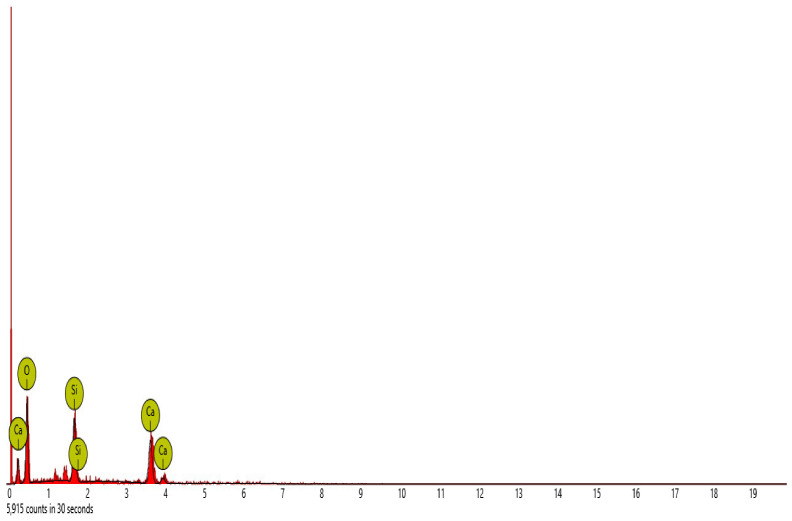
Graphical charts illustrating the atomic quantifications and the weights of the elements discovered from the EDS spectra of pervious concrete spot 1 sample Y_4_.

**Figure 19:**
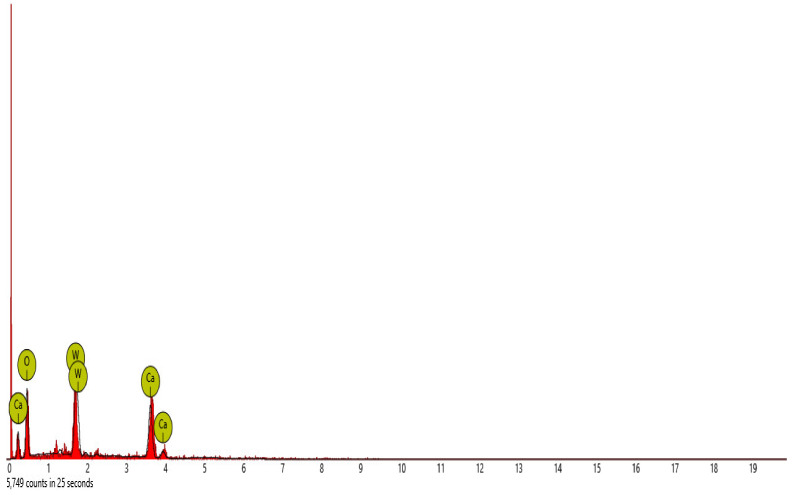
Bar charts illustration of the weight and atomic quantifications of the element discovered from the EDS spectra of pervious concrete spot 2 sample Y_4_.

Figure 15, Figure 16, Figure 17, Figure 18 and Figure 19 and Table 12, Table 13, Table 14, Table 15 and Table 16 present the EDS spectra of the test of pervious concrete samples which illustrates the fundamental analysis and works with SEM to provide the chemical composition and analysis of the test specimens blended with QD and SDA. From the EDS analysis, the mix 1 sample spot 1 portrayed a major peak composition at oxygen, calcium and silicon with atomic weights of 67.08, 24.16 and 8.76, respectively, and 25.91 of calcium, 64.67 of oxygen and 9.42 of bromine at spot 2. For mix 3, atomic weights of 20.17, 27.53, 31.88, 15.33 and 5.09 for tellurium, calcium, oxygen, silicon and strontium were used, respectively. Furthermore, mix 4 specimen spot 1 exhibited a major peak composition with atomic concentrations of 63.26, 23.24 and 13.50 for oxygen, calcium and silicon, respectively, while spot 2 portrayed 23.71, 29.04 and 47.25 for tungsten, calcium and oxygen, respectively. The increase in silicon, calcium and oxygen contents in the blended pervious concrete specimens could be due to a pozzolanic reaction in which the calcium from the SDA; and the cement chemically reacts with the silica and alumina from the QD, SDA and aggregates in the presence of water to produce stable calcium silicate hydrate and calcium aluminate hydrate, which results in long-term mechanical strength behavior [54,81].

## 4. Conclusions

The mechanical behavior of the green pervious concrete incorporated with solid waste materials and derivatives—namely SDA and QD—was investigated using Scheffe’s mixture theory to derive the optimum mixture concentration. The following conclusions were taken from the findings and outcomes of the research work:

- Using Scheffe’s optimization method, the flexural strength properties of the pervious concrete mixed with SDA and QD was modeled using a quadratic polynomial order. When the ratios are known, the created model may forecast the flexural strength of pervious concrete made up of five constituents, and vice versa.
- The permeability test, which offers a critical evaluation of the functional behavior of pervious concrete, revealed a maximum response of 7.32 mm/s at SDA and QD contents of 2.833% and 3.683% in the concrete, respectively, allowing surface runoff to permeate through it and be directed towards a safe discharge point to prevent flooding and erosion issues. The calculated findings revealed a decrease in the hydraulic conductivity of the concrete as the SDA and QD fractions increased, indicating the existence of more voids at lower SDA and QD contents of 1.5–3.0% and 3.2–3.7% in the mixture, respectively.
- The examination of the rheological characteristics of freshly mixed concrete shows that the inclusion of pozzolanic materials reduced the slump behavior of the fresh blended pervious concrete while increasing the setting time response to maximum with a cement–SDA blend ratio of 0.75:0.25.
- The maximum compressive strength obtained from the experimental program after 28 days of curing within the factor space was 3.703 N/mm^2^ with a mix proportion of 0.435:0.95:0.1:1.55:0.05 for water, cement, QD, coarse aggregate and SDA, respectively, and the minimum flexural strength response of 2.504 N/mm^2^ was obtained with a mix ratio of 0.6:0.75:0.3:4.1:0.25 for water, cement, QD, coarse aggregate and SDA, respectively.
- Using Student’s *t*-test and an analysis of variance, the model’s prediction ability was statistically evaluated and validated (ANOVA). There is no substantial discrepancy between the model-predicted and actual control results, according to the computed performance evaluation result. The obtained results for flexural strength, abrasion resistance and permeability response showed robust strength applications for road drainage purposes. Additionally, with the aid of scanning electron microscopy and electron diffraction spectroscopy, the pooled effects of SDA and QD in green pervious concrete production were qualitatively inveterate.
- Thus, the combination of QD and SDA aided the concrete matrix to form porous micro structures and enhanced its mechanical and hydraulic gradient properties. Finally, the added SDA and QD were observed to be valuable constituents in the development of the green pervious concrete material with saving costs through the recycling of solid wastes and its derivatives as well as enhanced its mechanical behavior and hydraulic gradient performance for pavement applications to successfully drain storm water.

## Figures and Tables

**Table 1:** Mixture Proportion Formulation.

Actual						Pseudo				
Z_1_	Z_2_	Z_3_	Z_4_	Z_5_	Response	X_1_	X_2_	X_3_	X_4_	X_5_
0.435	0.95	0.1	1.55	0.05	Y_1_	1	0	0	0	0
0.45	0.9	0.13	1.95	0.1	Y_2_	0	1	0	0	0
0.5	0.85	0.19	2.85	0.15	Y_3_	0	0	1	0	0
0.55	0.8	0.25	3.55	0.2	Y_4_	0	0	0	1	0
0.6	0.75	0.3	4.1	0.25	Y_5_	0	0	0	0	1
0.4425	0.925	0.115	1.75	0.075	Y_12_	0.5	0.5	0	0	0
0.4675	0.9	0.145	2.2	0.1	Y_13_	0.5	0	0.5	0	0
0.4925	0.875	0.175	2.55	0.125	Y_14_	0.5	0	0	0.5	0
0.5175	0.85	0.2	2.825	0.15	Y_15_	0.5	0	0	0	0.5
0.475	0.875	0.16	2.4	0.125	Y_23_	0	0.5	0.5	0	0
0.5	0.85	0.19	2.75	0.15	Y_24_	0	0.5	0	0.5	0
0.525	0.825	0.215	3.025	0.175	Y_25_	0	0.5	0	0	0.5
0.525	0.825	0.22	3.2	0.175	Y_34_	0	0	0.5	0.5	0
0.55	0.8	0.245	3.475	0.2	Y_35_	0	0	0.5	0	0.5
0.575	0.775	0.275	3.825	0.225	Y_45_	0	0	0	0.5	0.5

**Table 2:** Mixture Proportion Formulation for Control Points.

Actual						Pseudo				
Z_1_	Z_2_	Z_3_	Z_4_	Z_5_	Response	X_1_	X_2_	X_3_	X_4_	X_5_
0.48375	0.875	0.1675	2.475	0.125	C_1_	0.25	0.25	0.25	0.25	0
0.49625	0.8625	0.18	2.6125	0.1375	C_2_	0.25	0.25	0.25	0	0.25
0.50875	0.85	0.195	2.7875	0.15	C_3_	0.25	0.25	0	0.25	0.25
0.52125	0.8375	0.21	3.0125	0.1625	C_4_	0.25	0	0.25	0.25	0.25
0.525	0.825	0.2175	3.1125	0.175	C_5_	0	0.25	0.25	0.25	0.25
0.507	0.85	0.194	2.8	0.15	C_12_	0.2	0.2	0.2	0.2	0.2
0.4705	0.89	0.151	2.26	0.11	C_13_	0.3	0.3	0.3	0.1	0
0.4755	0.885	0.156	2.315	0.115	C_14_	0.3	0.3	0.3	0	0.1
0.4905	0.87	0.174	2.525	0.13	C_15_	0.3	0.3	0	0.3	0.1
0.5055	0.855	0.192	2.795	0.145	C_23_	0.3	0	0.3	0.3	0.1
0.51	0.84	0.201	2.915	0.16	C_24_	0	0.3	0.3	0.3	0.1
0.5385	0.815	0.232	3.305	0.185	C_25_	0.1	0	0.3	0.3	0.3
0.5235	0.83	0.214	3.035	0.17	C_34_	0.1	0.3	0	0.3	0.3
0.5085	0.845	0.196	2.825	0.155	C_35_	0.1	0.3	0.3	0	0.3
0.4935	0.86	0.181	2.66	0.14	C_45_	0.1	0.3	0.3	0.3	0

**Table 3:** Gradation Coefficients.

Test Materials	D_10_	D_30_	D_60_	C_u_	C_c_
SDA	0.06	0.085	0.125	2.083333	0.963333
Coarse Agg.	1.85	3	5.1	2.756757	0.953895
QD	0.135	0.4	0.9	6.666667	1.316872

**Table 4:** Chemical constituents of samples using X-ray fluorescence (XRF).

Oxide	CuO	Na_2_O	Fe_2_O_3_	MnO	Cr_2_O_3_	TiO_2_	CaO	Al_2_O_3_	MgO	ZnO	SO_3_	SiO_2_	LOI
SDA (%)	0.085	1.0	4.3	0.45	Nil	0.07	10.4	8.35	3.01	Nil	0.89	57.85	6.5
QD (%)	Nil	Nil	6.01	Nil	0.2	3.612	13.52	15.93	4.78	0.005	Nil	48.5	1.8

**Table 5:** Chemical properties of cement.

Oxide	CaO	MgO	Fe_2_O_3_	Na_2_O	Al_2_O_3_	SiO_2_	MnO	LOI	CUO	TiO_2_	CdO	K_2_O
Cement (%)	11.3	0.093	6.405	2.1	20.6	52.4	Trace	3.9	Trace	0.52	Trace	2.6

**Table 6:** Flexural strength response for the experimental points.

	Z_1_	Z_2_	Z_3_	Z_4_	Z_5_	Flex. Str. (N/mm^2^)	X_1_	X_2_	X_3_	X_4_	X_5_
Y_1_	0.435	0.95	0.1	1.55	0.05	3.7028	1	0	0	0	0
Y_2_	0.45	0.9	0.13	1.95	0.1	3.5303	0	1	0	0	0
Y_3_	0.5	0.85	0.19	2.85	0.15	3.606	0	0	1	0	0
Y_4_	0.55	0.8	0.25	3.55	0.2	3.2928	0	0	0	1	0
Y_5_	0.6	0.75	0.3	4.1	0.25	2.5036	0	0	0	0	1
Y_12_	0.4425	0.925	0.115	1.75	0.075	3.5936	0.5	0.5	0	0	0
Y_13_	0.4675	0.9	0.145	2.2	0.1	3.5012	0.5	0	0.5	0	0
Y_14_	0.4925	0.875	0.175	2.55	0.125	3.4508	0.5	0	0	0.5	0
Y_15_	0.5175	0.85	0.2	2.825	0.15	3.3036	0.5	0	0	0	0.5
Y_23_	0.475	0.875	0.16	2.4	0.125	3.4264	0	0.5	0.5	0	0
Y_24_	0.5	0.85	0.19	2.75	0.15	3.3252	0	0.5	0	0.5	0
Y_25_	0.525	0.825	0.215	3.025	0.175	3.2352	0	0.5	0	0	0.5
Y_34_	0.525	0.825	0.22	3.2	0.175	3.2456	0	0	0.5	0.5	0
Y_35_	0.55	0.8	0.245	3.475	0.2	3.1948	0	0	0.5	0	0.5
Y_45_	0.575	0.775	0.275	3.825	0.225	2.8232	0	0	0	0.5	0.5

**Table 7:** Flexural strength response for the control points.

	Z_1_	Z_2_	Z_3_	Z_4_	Z_5_	Flex. Str. (N/mm^2^)	X_1_	X_2_	X_3_	X_4_	X_5_
C_1_	0.484	0.875	0.168	2.475	0.125	3.6808	0.25	0.25	0.25	0.25	0
C_2_	0.496	0.863	0.180	2.613	0.138	3.5152	0.25	0.25	0.25	0	0.25
C_3_	0.509	0.850	0.195	2.788	0.150	3.4628	0.25	0.25	0	0.25	0.25
C_4_	0.521	0.838	0.210	3.013	0.163	3.434	0.25	0	0.25	0.25	0.25
C_5_	0.525	0.825	0.218	3.113	0.175	3.2896	0	0.25	0.25	0.25	0.25
C_12_	0.507	0.850	0.194	2.800	0.150	3.2528	0.2	0.2	0.2	0.2	0.2
C_13_	0.471	0.890	0.151	2.260	0.110	3.6504	0.3	0.3	0.3	0.1	0
C_14_	0.476	0.885	0.156	2.315	0.115	3.646	0.3	0.3	0.3	0	0.1
C_15_	0.491	0.870	0.174	2.525	0.130	3.5956	0.3	0.3	0	0.3	0.1
C_23_	0.506	0.855	0.192	2.795	0.145	3.298	0.3	0	0.3	0.3	0.1
C_24_	0.510	0.840	0.201	2.915	0.160	3.2636	0	0.3	0.3	0.3	0.1
C_25_	0.539	0.815	0.232	3.305	0.185	3.3308	0.1	0	0.3	0.3	0.3
C_34_	0.524	0.830	0.214	3.035	0.170	3.2832	0.1	0.3	0	0.3	0.3
C_35_	0.509	0.845	0.196	2.825	0.155	3.3656	0.1	0.3	0.3	0	0.3
C_45_	0.494	0.860	0.181	2.660	0.140	3.1928	0.1	0.3	0.3	0.3	0

**Table 8:** Flexural strength model coefficient.

β_1_	β_2_	β_3_	β_4_	β_5_	β_12_	β_13_	β_14_	β_15_	β_23_	β_24_	β_25_	β_34_	β_35_	β_45_
3.70	3.53	3.61	3.29	2.50	−0.09	−0.61	−0.19	0.80	−0.57	−0.35	0.87	−0.82	0.56	−0.30

**Table 9:** ANOVA test results.

Summary						
**Groups**	**Count**	**Sum**	**Average**	**Variance**		
Exp results	15	50.0348	3.335653	0.017872		
Predicted results	15	49.66066	3.310711	0.008116		
**Source of Variation**	**SS**	**df**	**MS**	**F**	***p*-value**	**F crit**
Between groups	0.004666	1	0.004666	0.359077	0.553837	4.195972
Within groups	0.363836	28	0.012994			
Total	0.368502	29				

**Table 10:** *T*-test for compressive strength properties.

	Exp. Results	Predicted Results
Mean	3.335653	3.310711
Variance	0.017872	0.008116
Observations	15	15
Pearson correlation	0.907144	
Degrees of freedom	14	
t Stat	1.50179	
P(T ≤ t) one-tail	0.077684	
t critical one-tail	1.76131	
P(T ≤ t) two-tail	0.155367	
t critical two-tail	2.144787	

**Table 11:** Sensitivity Analysis Computation.

S/n	Parameter Removed	MAE	RMSE	Rank
1	OPC	3.85	4.22	2
2	SDA	1.74	2.62	5
3	w/c	4.17	4.51	1
4	Coarse Agg.	1.96	2.97	4
5	QD	3.64	3.99	3

**Table 12:** SEM-EDS result for Y_1_ concrete sample spot 1.

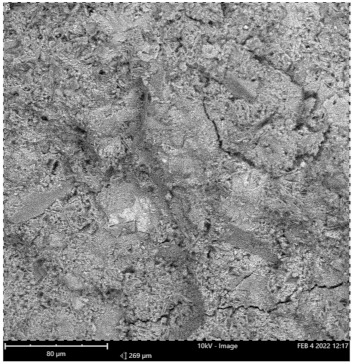	**Element** **Number**	**Element** **Symbol**	**Element** **Name**	**Atomic** **Conc.**	**Weight** **Conc.**
	8	O	Oxygen	67.08	46.91
	20	Ca	Calcium	24.16	42.33
	14	Si	Silicon	8.76	10.75

FOV: 269 µm; mode: 10 kV—image; detector: BSD Full; time: FEB 4 2022 12:17.

**Table 13:** SEM-EDS result for Y_1_ concrete sample spot 2.

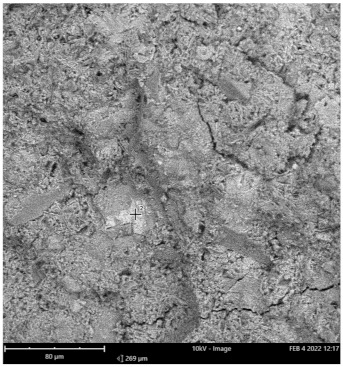	**Element** **Number**	**Element** **Symbol**	**Element** **Name**	**Atomic** **Conc.**	**Weight** **Conc.**
	20	Ca	Calcium	25.91	36.74
	8	O	Oxygen	64.67	36.62
	35	Br	Bromine	9.42	26.64

FOV: 269 µm; mode: 10 kV—image; detector: BSD Full; time: FEB 4 2022 12:17.

**Table 14:** SEM-EDS result for Y_3_ concrete sample spot.

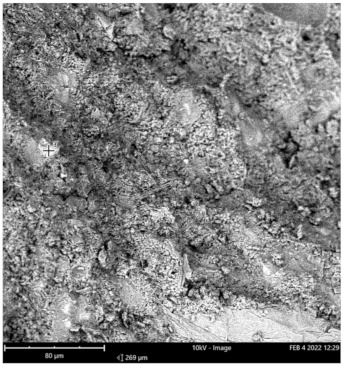	**Element** **Number**	**Element** **Symbol**	**Element** **Name**	**Atomic** **Conc.**	**Weight** **Conc.**
	52	Te	Tellurium	20.17	50.83
	20	Ca	Calcium	27.53	21.79
	8	O	Oxygen	31.88	10.07
	38	Sr	Strontium	5.09	8.80
	14	Si	Silicon	15.33	8.50

FOV: 269 µm; mode: 10 kV—image; detector: BSD Full; time: FEB 4 2022 12:29.

**Table 15:** SEM-EDS result for Y_4_ concrete sample spot 1.

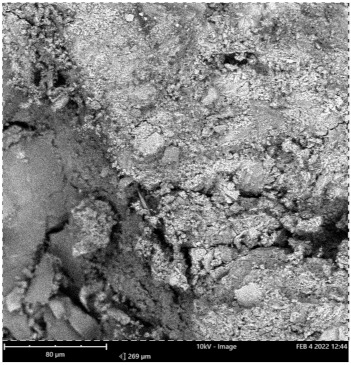	**Element** **Number**	**Element** **Symbol**	**Element** **Name**	**Atomic** **Conc.**	**Weight** **Conc.**
	8	O	Oxygen	63.26	43.57
	20	Ca	Calcium	23.24	40.10
	14	Si	Silicon	13.50	16.33

FOV: 269 µm; mode: 10 kV—image; detector: BSD Full; time: FEB 4 2022 12:44.

**Table 16:** SEM-EDS result for Y_4_ concrete sample spot 2.

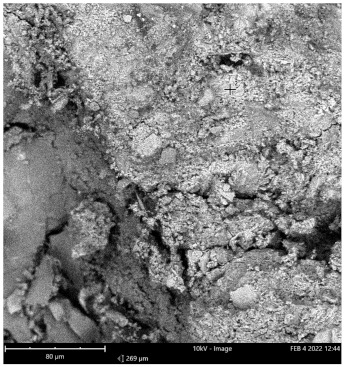	**Element** **Number**	**Element** **Symbol**	**Element** **Name**	**Atomic** **Conc.**	**Weight** **Conc.**
	74	W	Tungsten	23.71	69.43
	20	Ca	Calcium	29.04	18.53
	8	O	Oxygen	47.25	12.04

FOV: 269 µm; mode: 10 kV—image; detector: BSD Full; time: FEB 4 2022 12:44.

## Data Availability

Data supporting reported results is in the manuscript.

## Supplementary Materials

The following supporting information can be downloaded at: https://www.mdpi.com/article/10.3390/ma16020598/s1.
