# Supplementary material for: Scheffe’s Simplex Optimization of Flexural Strength of Quarry Dust and Sawdust Ash Pervious Concrete for Sustainable Pavement Construction
Source: Materials (Basel). 2023 Jan 7;16(2):598. doi: 10.3390/ma16020598 (PMC9863484; doi:10.3390/ma16020598)
Supplement: Supplementary file 1 [file materials-16-00598-s001.zip › materials-1984882-supplementary.pdf]

materials
MDPI

Surpporting information

Scheffe's Simplex Optimization of Flexural Strength of Quarry Dust and Sawdust Ash Pervious Concrete for Sustainable Pavement Construction

Flexural strength MATLAB Program

```
syms X1 X2 X3 X4 X5
Z1=[0.435 0.95 0.1 1.55 0.05];
Z2=[0.45 0.9 0.13 1.95 0.1];
Z3=[0.5 0.85 0.19 2.85 0.15];
Z4=[0.55 0.8 0.25 3.55 0.2];
Z5=[0.6 0.75 0.3 4.1 0.25];
A=[Z1' Z2' Z3' Z4' Z5'];
disp('matrix A =');
disp(A)

%PSEUDO COMPONENTS
x1=[1 0 0 0 0];
x2=[0 1 0 0 0];
x3=[0 0 1 0 0];
x4=[0 0 0 1 0];
x5=[0 0 0 0 1];
X12=[0.5 0.5 0 0 0];
X13=[0.5 0 0.5 0 0];
X14=[0.5 0 0 0.5 0];
X15=[0.5 0 0 0 0.5];
X23=[0 0.5 0.5 0 0];
X24=[0 0.5 0 0.5 0];
X25=[0 0.5 0 0 0.5];
X34=[0 0 0.5 0.5 0];
X35=[0 0 0.5 0 0.5];
X45=[0 0 0 0.5 0.5];

%PSEUDO COMPONENTS CONTROL
C1=[0.25 0.25 0.25 0.25 0];
C2=[0.25 0.25 0.25 0 0.25];
C3=[0.25 0.25 0 0.25 0.25];
C4=[0.25 0 0.25 0.25 0.25];
C5=[0 0.25 0.25 0.25 0.25];
C12=[0.2 0.2 0.2 0.2 0.2];
C13=[0.3 0.3 0.3 0.1 0];
C14=[0.3 0.3 0.3 0 0.1];
C15=[0.3 0.3 0 0.3 0.1];
C23=[0.3 0 0.3 0.3 0.1];
C24=[0 0.3 0.3 0.3 0.1];
C25=[0.1 0 0.3 0.3 0.3];
C34=[0.1 0.3 0 0.3 0.3];
C35=[0.1 0.3 0.3 0 0.3];
C45=[0.1 0.3 0.3 0.3 0];
```

```
A12=A*X12';
A13=A*X13';
A14=A*X14';
A15=A*X15';
A23=A*X23';
A24=A*X24';
A25=A*X25';
A34=A*X34';
A35=A*X35';
A45=A*X45';

CT1=A*C1';
CT2=A*C2';
CT3=A*C3';
CT4=A*C4';
CT5=A*C5';
CT12=A*C12';
CT13=A*C13';
CT14=A*C14';
CT15=A*C15';
CT23=A*C23';
CT24=A*C24';
CT25=A*C25';
CT34=A*C34';
CT35=A*C35';
CT45=A*C45';

table=[Z1; Z2; Z3;Z4; Z5; A12';A13';A14';A15';A23';A24';A25';A34';A35';A45'];
disp('5,2 scheffe ratio')
disp(table)

control=[CT1'; CT2'; CT3';CT4'; CT5';
CT12';CT13';CT14';CT15';CT23';CT24';CT25';CT34';CT35';CT45'];
disp('control scheffe ratio')
disp(control)

% RESPONSE (COMPRESSIVE STRENGTH)
Y1=3.7028;
Y2=3.53032;
Y3=3.606;
Y4=3.2928;
Y5=2.5036;
Y12=3.5936;
Y13=3.5012;
Y14=3.4508;
Y15=3.3036;
Y23=3.4264;
Y24=3.3252;
Y25=3.2352;
Y34=3.2456;
Y35=3.1948;
Y45=2.8232;
```

```
%CONTROL RESPONSE
ct1=3.40;
ct2=3.39;
ct3=3.30;
ct4=3.23;
ct5=3.20;
ct12=3.21;
ct13=3.53;
ct14=3.65;
ct15=3.40;
ct23=3.38;
ct24=3.26;
ct25=3.11;
ct34=3.28;
ct35=3.37;
ct45=3.31;

% MODEL RELATIONSHIP
B1=Y1;
B2=Y2;
B3=Y3;
B4=Y4;
B5=Y5;
B12=4*Y12-2*Y1-2*Y2;
B13=4*Y13-2*Y1-2*Y3;
B14=4*Y14-2*Y1-2*Y4;
B15=4*Y15-2*Y1-2*Y5;
B23=4*Y23-2*Y2-2*Y3;
B24=4*Y24-2*Y2-2*Y4;
B25=4*Y25-2*Y2-2*Y5;
B34=4*Y34-2*Y3-2*Y4;
B35=4*Y35-2*Y3-2*Y5;
B45=4*Y45-2*Y4-2*Y5;
coefficient1=double([B1 B2 B3 B4 B5 B12 B13 B14 B15 B23 B24 B25 B34 B35 B45]');
y=(B1*X1)+(B2*X2)+(B3*X3)+(B4*X4)+(B5*X5)+(B12*X1*X2)+(B13*X1*X3)+(B14*X1*X4
)+(B15*X1*X5)+(B23*X2*X3)+(B24*X2*X4)+(B25*X2*X5)+(B34*X3*X4)+(B35*X3*X5)+(B45*X
4*X5);
disp('y =')
disp (y)
disp('coefficient')
disp(coefficient1)

M1=ct1;
M2=ct2;
M3=ct3;
M4=ct4;
M5=ct5;
M12=4*ct12-2*ct1-2*ct2;
M13=4*ct13-2*ct1-2*ct3;
M14=4*ct14-2*ct1-2*ct4;
M15=4*ct15-2*ct1-2*ct5;
M23=4*ct23-2*ct2-2*ct3;
M24=4*ct24-2*ct2-2*ct4;
M25=4*ct25-2*ct2-2*ct5;
M34=4*ct34-2*ct3-2*ct4;
```

```
M35=4*ct35-2*ct3-2*ct5;
M45=4*ct45-2*ct4-2*ct5;
coefficient2=double([M1 M2 M3 M4 M5 M12 M13 M14 M15 M23 M24 M25 M34 M35
M45]');
m=(M1*X1)+(M2*X2)+(M3*X3)+(M4*X4)+(M5*X5)+(M12*X1*X2)+(M13*X1*X3)+(M14
*X1*X4)+(M15*X1*X5)+(M23*X2*X3)+(M24*X2*X4)+(M25*X2*X5)+(M34*X3*X4)+(M35*X3
*X5)+(M45*X4*X5);

y1=subs(y,[X1 X2 X3 X4 X5],x1);
y2=subs(y,[X1 X2 X3 X4 X5],x2);
y3=subs(y,[X1 X2 X3 X4 X5],x3);
y4=subs(y,[X1 X2 X3 X4 X5],x4);
y5=subs(y,[X1 X2 X3 X4 X5],x5);
y12=subs(y,[X1 X2 X3 X4 X5],X12);
y13=subs(y,[X1 X2 X3 X4 X5],X13);
y14=subs(y,[X1 X2 X3 X4 X5],X14);
y15=subs(y,[X1 X2 X3 X4 X5],X15);
y23=subs(y,[X1 X2 X3 X4 X5],X23);
y24=subs(y,[X1 X2 X3 X4 X5],X24);
y25=subs(y,[X1 X2 X3 X4 X5],X25);
y34=subs(y,[X1 X2 X3 X4 X5],X34);
y35=subs(y,[X1 X2 X3 X4 X5],X35);
y45=subs(y,[X1 X2 X3 X4 X5],X45);

%control equation substitution
m1=subs(m,[X1 X2 X3 X4 X5],C1);
m2=subs(m,[X1 X2 X3 X4 X5],C2);
m3=subs(m,[X1 X2 X3 X4 X5],C3);
m4=subs(m,[X1 X2 X3 X4 X5],C4);
m5=subs(m,[X1 X2 X3 X4 X5],C5);
m12=subs(m,[X1 X2 X3 X4 X5],C12);
m13=subs(m,[X1 X2 X3 X4 X5],C13);
m14=subs(m,[X1 X2 X3 X4 X5],C14);
m15=subs(m,[X1 X2 X3 X4 X5],C15);
m23=subs(m,[X1 X2 X3 X4 X5],C23);
m24=subs(m,[X1 X2 X3 X4 X5],C24);
m25=subs(m,[X1 X2 X3 X4 X5],C25);
m34=subs(m,[X1 X2 X3 X4 X5],C34);
m35=subs(m,[X1 X2 X3 X4 X5],C35);
m45=subs(m,[X1 X2 X3 X4 X5],C45);

table2=double([y1 y2 y3 y4 y5 y12 y13 y14 y15 y23 y24 y25 y34 y35 y45]');
disp('model response')
disp(table2)

table3=double([m1 m2 m3 m4 m5 m12 m13 m14 m15 m23 m24 m25 m34 m35 m45]');
disp('model control response')
disp(table3)

%trial test run
Test=[0.1 0.4 0.2 0.2 0.1];
double(subs(y,[X1 X2 X3 X4 X5],Test))

Ans
```

3.3354
